# Inferring the distribution of fitness effects of spontaneous mutations in *Chlamydomonas reinhardtii*

**DOI:** 10.1371/journal.pbio.3000192

**Published:** 2019-06-26

**Authors:** Katharina B. Böndel, Susanne A. Kraemer, Toby Samuels, Deirdre McClean, Josianne Lachapelle, Rob W. Ness, Nick Colegrave, Peter D. Keightley

**Affiliations:** 1 Institute of Evolutionary Biology, Ashworth Laboratories, University of Edinburgh, Edinburgh, United Kingdom; 2 Biology Department, Concordia University, Montreal, Quebec, Canada; 3 Institute of Immunology and Infection Research, Ashworth Laboratories, University of Edinburgh, Edinburgh, United Kingdom; 4 Department of Biology, William G. Davis Building, University of Toronto, Mississauga, Canada; Fred Hutchinson Cancer Research Center, UNITED STATES

## Abstract

Spontaneous mutations are the source of new genetic variation and are thus central to the evolutionary process. In molecular evolution and quantitative genetics, the nature of genetic variation depends critically on the distribution of effects of mutations on fitness and other quantitative traits. Spontaneous mutation accumulation (MA) experiments have been the principal approach for investigating the overall rate of occurrence and cumulative effect of mutations but have not allowed the phenotypic effects of individual mutations to be studied directly. Here, we crossed MA lines of the green alga *Chlamydomonas reinhardtii* with its unmutated ancestral strain to create haploid recombinant lines, each carrying an average of 50% of the accumulated mutations in a large number of combinations. With the aid of the genome sequences of the MA lines, we inferred the genotypes of the mutations, assayed their growth rate as a measure of fitness, and inferred the distribution of fitness effects (DFE) using a Bayesian mixture model. We infer that the DFE is highly leptokurtic (L-shaped). Of mutations with absolute fitness effects exceeding 1%, about one-sixth increase fitness in the laboratory environment. The inferred distribution of effects for deleterious mutations is consistent with a strong role for nearly neutral evolution. Specifically, such a distribution predicts that nucleotide variation and genetic variation for quantitative traits will be insensitive to change in the effective population size.

## Abstract 2

Recombinant lines of the green alga *Chlamydomonas reinhardtii* containing combinations of spontaneously accumulated mutations allowed researchers to infer the distribution of effects of individual mutations on fitness from the genotypic plus phenotypic data.

## Introduction

Understanding evolution requires an understanding of the origin of new genetic variation from mutation, including the rates of mutation at individual loci and the magnitudes of their effects on fitness and other traits. Of particular interest is the distribution of fitness effects (DFE) for new mutations, describing the relative rates of occurrence of mutations with different selective effect sizes. The DFE informs about the frequencies of small- versus large-effect mutations and the frequencies of advantageous versus deleterious mutations and is therefore of fundamental importance in population and quantitative genetics. For example, the DFE appears in the nearly neutral model of molecular evolution [1], in which deleterious mutations are effectively selected against in large populations but behave as selectively neutral in small populations. Kimura [2,3] showed that if the DFE is strongly leptokurtic (i.e., L-shaped with most of the density concentrated near zero and a long tail of mutations with increasing deleterious effects), molecular genetic variation at sites subject to natural selection increases slowly with increasing effective population size (*N*_*e*_), and molecular evolution is potentially clocklike between species with different effective size. This is therefore broadly consistent with empirical observations. The DFE is also important for predicting selection response for quantitative traits and the nature of quantitative genetic variation [4]. For example, the contribution of mutation to response to selection depends critically on the shape of the DFE, the response occurring more quickly, on average, with more leptokurtic distributions [5]. Analogously with the relationship between nucleotide variation and *N*_*e*_, genetic variation for fitness (or a trait correlated with fitness) is predicted to increase slowly as a function of *N*_*e*_ if the DFE is leptokurtic [6] and could thus explain why genetic variation for quantitative traits is apparently relatively invariant between species [7].

In the light of its fundamental importance, there has been much previous work aimed at inferring the DFE. Two different approaches have principally been applied for spontaneous mutations occurring in the whole genome (rather than just in a single locus): the analysis of nucleotide polymorphism data from individuals sampled from a population and spontaneous mutation accumulation (MA) experiments [8]. Under the former approach [9–12], the site frequency spectra for putatively neutral and selected sites (typically synonymous and nonsynonymous sites of protein-coding genes, respectively) are compared and parameters of the DFE for the mutations at the selected sites inferred. The approach makes several assumptions, notably that variation at the selected sites is explained by a balance between an input of new deleterious mutations, natural selection, and genetic drift and that selection is absent from the putatively neutral class of sites. It is only capable of inferring the DFE for mutations that stand an appreciable chance of segregating in the sample of individuals from the population, implying that inferences are only relevant to mutations with effects that are not substantially greater than 1/*N*_*e*_. This can be an extremely small value if *N*_*e*_ is large. Furthermore, it can only be applied to specific functional categories of sites in the genome.

In a spontaneous MA experiment, sublines of the same initial genotype are maintained at small effective population size in the near absence of natural selection for many generations, allowing mutations to accumulate effectively at random. The DFE can be estimated using the among-MA line distribution of phenotypic values for traits related to fitness (such as fecundity or viability) [13–15]. The information that can be obtained on the DFE by this approach is extremely limited, however, principally because the numbers of mutations carried by individual lines are not included in the analysis, so an overall genomic rate parameter has to be estimated, and this is highly confounded with the DFE parameters [16,17].

Genome sequencing technology now allows the identification of the nearly complete complement of mutations carried by a set of MA lines, and in combination with phenotypic information this can potentially be used to leverage information on the DFE [18]. Previous analysis of spontaneous MA experiments have, however, only studied the cumulative effects of new mutations, whereas accurate inference is likely to require a model that includes the effects of individual mutations. For example, we have shown that there is a negative correlation between the number of new mutations carried by a MA line and fitness, but this gives only limited information on the DFE [19].

Previously, we carried out a spontaneous MA experiment in the single-celled green alga *Chlamydomonas reinhardtii* for approximately 1,000 generations, have measured fitness-related traits in a range of environmental conditions [19–21], and have employed genome sequencing to determine the complement of mutations carried by the lines [22]. Here, we have crossed six of these *C*. *reinhardtii* MA lines of the CC-2931 genetic background with a compatible ancestor of the same background genotype but of the opposite mating type. We thereby generated 1,526 recombinant lines (RLs), each carrying an average of 50% of the mutations of the MA line parent in different combinations. We genotyped the RLs at the locations of the known mutations and assayed their growth rate, a trait that is strongly correlated with competitive fitness in laboratory assays [19]. Across the six lines, there are nearly 400 unique mutations, so an analysis in which each mutation is treated as a fixed effect is not appropriate. Instead, we developed a Markov chain Monte Carlo (MCMC) approach with a random-effects model in which mutation effects are assumed to be sampled from some distribution or a mixture of distributions. We investigate a number of distributions to infer the distribution of effects for the individual mutations on growth rate. We show that the DFE is highly leptokurtic (L-shaped) and that an appreciable proportion of mutations increase fitness in the laboratory environment.

## Results

To directly infer the DFE, we crossed six *C*. *reinhardtii* MA lines derived from the CC-2931 strain to an ancestral strain of the same genetic background and the opposite mating type to produce a total of 1,526 RLs (Table 1, S1 Table). We genotyped 386 of the 476 mutations detected in our previous whole-genome sequencing study in these lines [22] (Table 1, S2 Table). Among the 681 different recovered haplotypes, mutations were present at an average frequency of 49.1% (10.3%–85.4%), which is close to the expected average of 50% (S1 Fig). The number of haplotypes obtained for each MA line and their frequencies was quite variable, however (S3 Table). For example, we obtained 214 haplotypes for MA line L03, and no haplotype was found more than four times, whereas we obtained only 67 haplotypes for MA line 14, and one of these haplotypes was found 18 times.

**Table 1:** Data overview.

MA line cross	Number of mutations	Number of RLs	Number of haplotypes
L03	39	247	214
L06	69	238	109
L07	59	261	69
L09	98	272	68
L11	66	272	154
L14	55	236	67
Combined	386	1,526	681

Abbreviations: MA, mutation accumulation; RL, recombinant line.

### Relationship between number of mutations and growth rate

**Fig 1:**
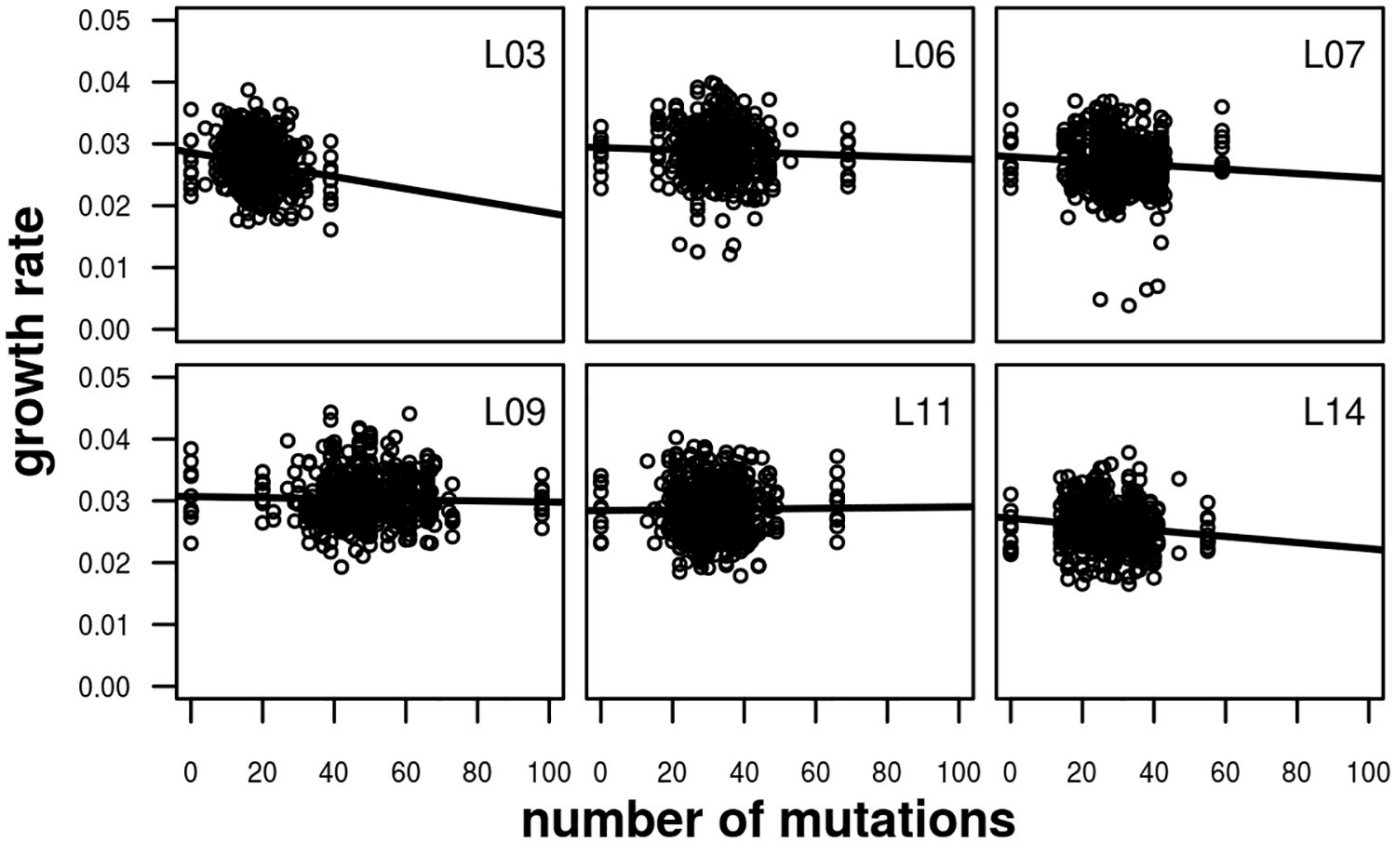
Relationship between growth rate and number of mutations carried by an RL or ancestor for the six CC-2931 MA line crosses. Linear regression lines are shown. MA, mutation accumulation; RL, recombinant line.

As a measure of fitness, we assayed the maximum growth rate of each RL, the parental MA lines, and the unmutated ancestral strain in liquid culture. To determine whether mutations have an overall directional effect on fitness, we used mixed models to test for a relationship between the number of mutations carried by RLs and their ancestors and fitness (Fig 1). In the case of only one of the six MA lines (L03), including number of mutations led to a significantly better fit (*P* = 0.0008; Table 2), and the improvement in fit for an analysis of the combined data set of all six MA line crosses was nonsignificant (*P* = 0.080). This could either mean that there is insufficient power to detect mutational effects or that there is a mixture of mutations with positive and negative effects on fitness. The latter explanation is supported, because there is a highly significant between-haplotype component of variation for the trait (*P* < 2.2 × 10^−16^ for the whole data set; *P* between 4.1 × 10^−13^ [L14] and 0.022 [L06] for the individual MA lines). We repeated this analysis fitting number of mutations of specific types (SNP, indel, exonic, intronic, intergenic; S4 Table). Including the number of mutations gave a significantly better fit in the cases of MA line L03 for all mutation types except intronic, for MA line L11 for intronic mutations, and for the whole data for exonic mutations.

**Table 2:** Likelihood ratio tests for mixed-model analysis of growth rate as a function of number of mutations of all kinds with 1 degree of freedom.

MA line cross	Chi square	*P* value
L03	11.2	0.00080
L06	0.77	0.38
L07	0.18	0.67
L09	0.072	0.79
L11	2.4	0.12
L14	0.72	0.40
Whole data set	3.1	0.080

Abbreviation: MA, mutation accumulation.

### Inference of the DFE by MCMC assuming mutation effects fall into discrete categories

The relationship between mutation number and fitness tells us little about the DFE for individual mutations. We therefore developed an approach that allows posterior distributions of the DFE parameters to be obtained in a Bayesian mixture model (implemented by MCMC). This assumes that the effects of mutations come from either a mixture of point masses or a mixture of gamma distributions. To maximize power, we focussed much of the analysis on a merged data set of all six MA line backcrosses.

We first examined whether there is evidence for an overall directional effect of new mutations on growth rate by running the analysis while assuming a two-category model with one nonzero effect category (effect = *e*_1_, proportion = *q*_1_) and one zero-effect category (i.e., *e*_0_ = 0, *q*_0_ = 1 − *q*_1_). The results (Table 3, S2 and S3 Figs) suggest that there is an appreciable frequency (approximately 4%) of mutations reducing growth rate by approximately 3%, whereas the majority of mutations are allocated to the zero-effect category.

**Table 3:** Bayesian MCMC estimates based on modes of the posterior distributions and 95% credible intervals for mutation effect (*e*) and mutation frequency (*q*) parameters under two- or three-category models along with BIC relative to the model with two categories of mutation effects. Both models include a class of mutations with zero effect on the trait.

	Parameter estimate (95% credible interval)				
Model (no. mutation categories)	e_1_	q_1_	e_2_	q_2_	BIC
2	−0.031 (−0.044, −0.023)	0.042 (0.020, 0.079)	-	-	0
3	−0.024 (−0.043, −0.011)	0.071 (0.031, 0.42)	0.021 (0.010, 0.068)	0.048 (0.010, 0.41)	−147

Abbreviations: BIC, Bayesian information criterion; MCMC, Markov chain Monte Carlo.

We then analysed the combined data set assuming a model with three categories of mutational effects (one zero-effect category and two finite-effect categories, *e*_1_ and *e*_2_). As expected, given that the two-category model supports the presence of negative mutational effects, a category of negative effects is inferred (*e*_1_, Table 3; S4 and S5 Figs). This has a similar posterior mode as the two-category model, but the credible interval is somewhat wider, as expected for a more parameter-rich model. There is also support for a class of positive-effect mutations (*e*_2_), which has a somewhat lower absolute modal estimate than the negative category. The estimated frequency of positive-effect mutations is lower than that of the negative-effect mutations. Based on the Bayesian information criterion (BIC), there is very strong evidence in favour of the three-category model over the two-category model. Results from the analysis of data from individual MA line crosses (S5 Table) are consistent with the presence of a mixture of negative- and positive-effect mutations.

We then analysed data sets in which phenotypic values were permuted within plate. As expected under the null model, the distributions of estimated values of *e*_1_ and *e*_2_ centre on zero, and the estimates of *e*_1_ and *e*_2_ from the real data are well outside the distributions obtained from permuted data (S6 Fig).

Analysis of a model with four categories of effects (one of which is a zero-effect category) also gives negative and positive posterior modes for two classes of mutational effects *e*_1_ and *e*_2_. However, it is difficult to determine whether there is an additional mutational class *e*_*3*_ that is different from the zero-effect class or *e*_1_ and *e*_2_ because of the presence of label switching [23] between the three classes of effects and their frequencies.

### Two-sided gamma DFE model

**Fig 2:**
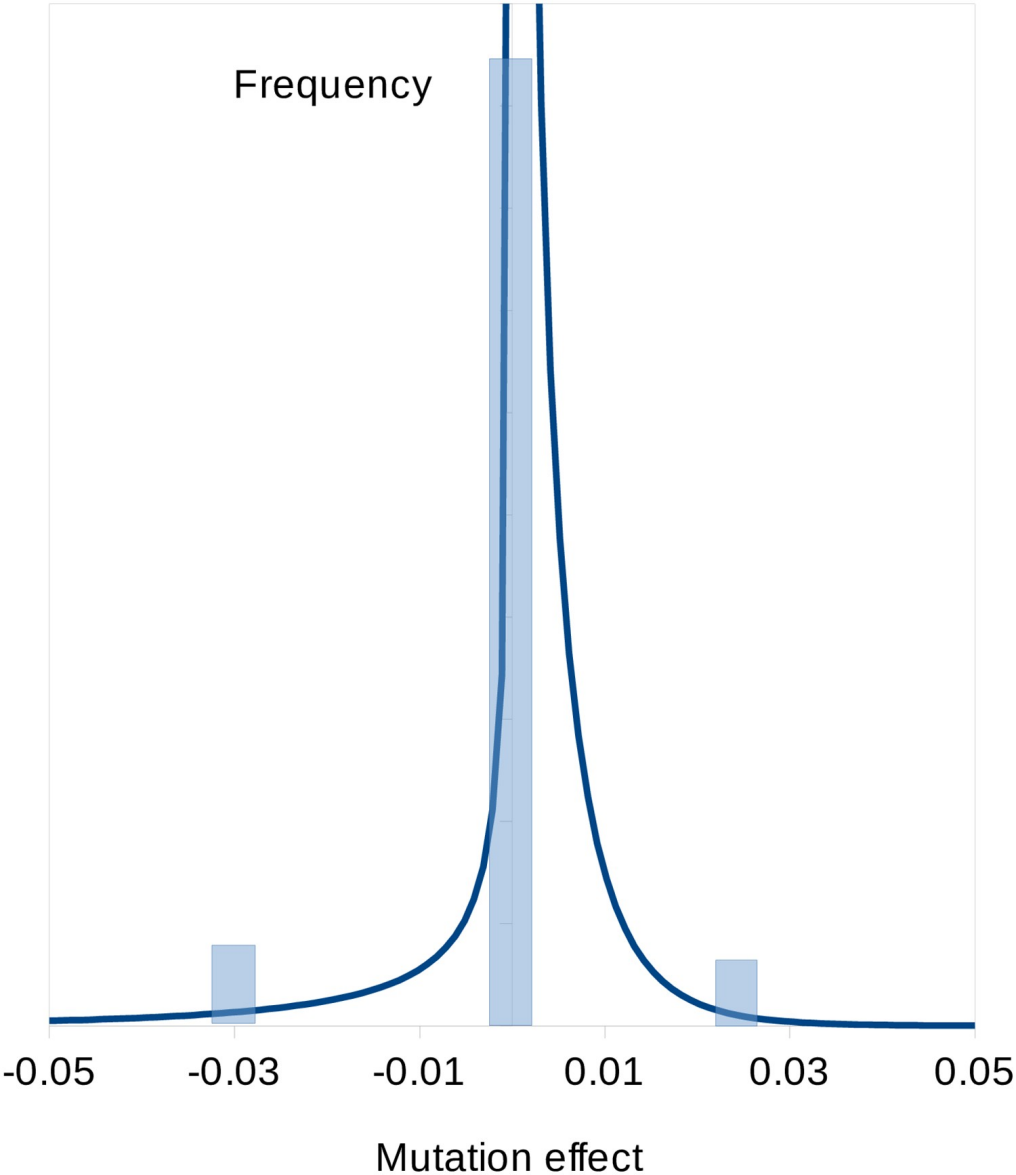
Inferred DFE assuming a two-sided gamma model (smooth line) and a point mass DFE for the three-category model (transparent blue rectangles). DFE, distribution of fitness effects.

Although informative about the overall directional effects of mutations, models in which mutations fall into discrete categories are unrealistic, because they assume no variance among the effects of mutations within each category. We therefore analysed the combined data set for the six MA line crosses under a two-sided gamma distribution of effects, which assumes that the effects of mutations are continuously distributed. We assumed the gamma distribution, because it is a flexible two-parameter distribution (*α* = scale, *β* = shape) that can take a wide variety of shapes, ranging from a highly leptokurtic, L-shaped distribution (*β* → 0) to a point mass (*β* → ∞). We analysed a model in which positive- and negative-effect mutations can have either the same or different absolute means, but their distributions have the same shape parameter. The results from the analysis of the combined data set (Table 4; S7 Fig) suggest that the DFE is highly leptokurtic (i.e., *β* is close to 0.3) and that the means for positive- and negative-effect mutations (= *β*/*α*) are very small, reflecting the concentration of mutations with effects close to zero. Consistent with the analysis assuming discrete classes of mutations (Table 3), there is a substantial proportion of positive-effect mutations (i.e., approximately 80%; Table 4). Two-sided gamma distribution models (with the same or different means) are strongly favoured over the model with three discrete classes of mutations, including a zero-effect class (BIC = −1,340 and −1,740, respectively). The estimated DFE for the two-sided gamma distribution is shown along with that for the three-category point mass DFE in Fig 2.

**Table 4:** Bayesian estimates obtained from the modes of the posterior distributions and 95% credible intervals for parameters of gamma distributions of negative and positive mutation effects (indexed by 0 and 1, respectively), under two-sided gamma distribution models with the same or different means for negative- and positive-effect mutations. For example, *e*_1_ is the estimated mean of the gamma distribution of positive-effect mutations, and *q*_1_ is their frequency.

Parameter	Model	Estimate	95% credible interval	
β	Two-sided gamma, same means	0.32	0.26	0.70
*e*		0.0049	0.0037	0.0070
*q*_1_		0.48	0.39	0.58
β	Two-sided gamma, different means	0.30	0.24	0.71
*e*_0_		−0.0092	−0.020	−0.0060
*e*_1_		0.0021	0.0013	0.0032
*q*_1_		0.84	0.73	0.90

The credible intervals for the absolute means of negative- and positive-effect mutations do not overlap (Table 4), suggesting that the model with different means fits better than a model assuming a two-sided gamma distribution with the same means (Table 4; S8 Fig). A model with different shape parameters for negative- and positive-effect mutations gives similar estimates for the mean effects and proportion of positive-effect mutations as the model with a single shape parameter, but stable estimates of the shape parameters could not be obtained, suggesting that this model is overparameterised.

### Relationships between estimated mutation effects and mutation types

To investigate whether mutations in certain mutation classes (such as exonic/nonexonic) are more or less likely to be associated with fitness, we calculated the effect of each mutation (as the posterior mean) under the two-sided gamma distribution model and then computed the difference between the average squared effects for mutations in mutually exclusive annotation classes. We examined average squared differences, because the additive variance contributed by a mutation is proportional to its squared effect. The results are negative in the sense that there are no statistically significant relationships for any of the mutation types tested (Table 5).

**Table 5:** Average squared effects of mutations (×1,000) of certain mutation type classifications (S1 Data) estimated under the two-sided gamma distribution model. For example, in the row labelled ‘SNP versus Indel’, e^2^(−) and e^2^(+) are the average squared effects for SNP and indel mutations, respectively. *P* values for the difference between the squared effects of mutations were obtained by bootstrapping mutations 1,000 times.

Mutation type	e^2^(−)	e^2^(+)	*P* value
SNP versus indel	0.074	0.073	0.84
Nonexonic versus exonic	0.061	0.083	0.15
Nonintronic versus intronic	0.081	0.059	0.17
Nonintergenic versus intergenic	0.074	0.067	0.92

## Discussion

In this paper, we integrate information on the fitness of MA lines, ancestral lines, and crosses between MA lines and their ancestors with the complement of mutations carried by each line or cross. By crossing MA lines with their ancestors, each RL is expected to contain a different complement of mutations, which can be determined by genotyping. If there is sufficient replication, it is possible to estimate the individual phenotypic effects of mutations. The total number of mutations genotyped in the six MA lines studied was 386, however, implying that the effects of most mutations must be very small, and estimation of a fixed effect for each mutation is inappropriate. We therefore developed a random-effects model, fitted a mixture of distributions using MCMC, and obtained Bayesian estimates from modes of the posterior distributions of the parameters of the DFE. We investigated models in which each mutation is assigned to one of a number of classes of fitness effects (which includes a class with zero effect) or we assume that mutation effects are drawn from a mixture of gamma distributions. Our approach has similarities to the Bayesian mixture model method BayesR [24] developed to estimate the distribution of SNP effects in genome-wide association studies. BayesR simultaneously analyses all informative SNPs (we likewise include all mutations) and fits a mixture of distributions of SNP effects, including a zero-effect class. Specifically, BayesR estimates the relative frequencies of the zero-effect class and a mixture of normal distributions of SNP effects with fixed variances. In this respect, BayesR differs from our method, in which we estimate discrete categories of effects or gamma distribution parameters as variables in the model, and we also simultaneously estimate the frequencies of mutations in the different effects categories or gamma distributions.

Previous approaches to infer the DFE for spontaneous mutations using data from MA experiments have compared the distributions of estimated trait values for MA lines and unmutated controls. The simplest approach is the Bateman-Mukai method [25,26], which uses the changes of trait mean and genetic variance between MA lines and unmutated controls to estimate a genomic mutation rate parameter (*U*, the frequency of mutations with an effect on the trait) and the average effect of a mutation (*e*), while assuming that all mutations have the same effect. The information that can be obtained by the Bateman-Mukai method, and other approaches that use the full distribution of MA line phenotypic values [13,15], is extremely limited, however [17]. The limitation arises because the genomic mutation rate and *e* are confounded with one another under the Bateman-Mukai approach, so the DFE and *U* are also confounded, and there is little information to distinguish between alternative models for the DFE if the effects of mutations are assumed to vary [16].

For five of the six CC-2931 MA line crosses, there is a negative relationship between growth rate and the number of mutations carried by an RL, although in some cases the relationship is very weak. This result is broadly consistent with the tendency for most *C*. *reinhardtii* MA lines to have a lower growth rate than their ancestors [20,21] and with Kraemer and colleagues [19], who generally observed negative relationships between fitness measured in competition with a marked strain and the numbers of mutations carried for MA lines of several genetic backgrounds. Kraemer and colleagues [19] also attempted to estimate a multicategory DFE based on the relationship between mutation number and fitness, but the amount of information available was limited, principally because there were only 10–14 MA lines tested of each genetic background. Here, we have characterized 1,526 RLs and a large number of combinations of genotypes and therefore expect this design to be more powerful for inferring properties of the DFE than previous approaches that analysed individual MA lines.

**Fig 3:**
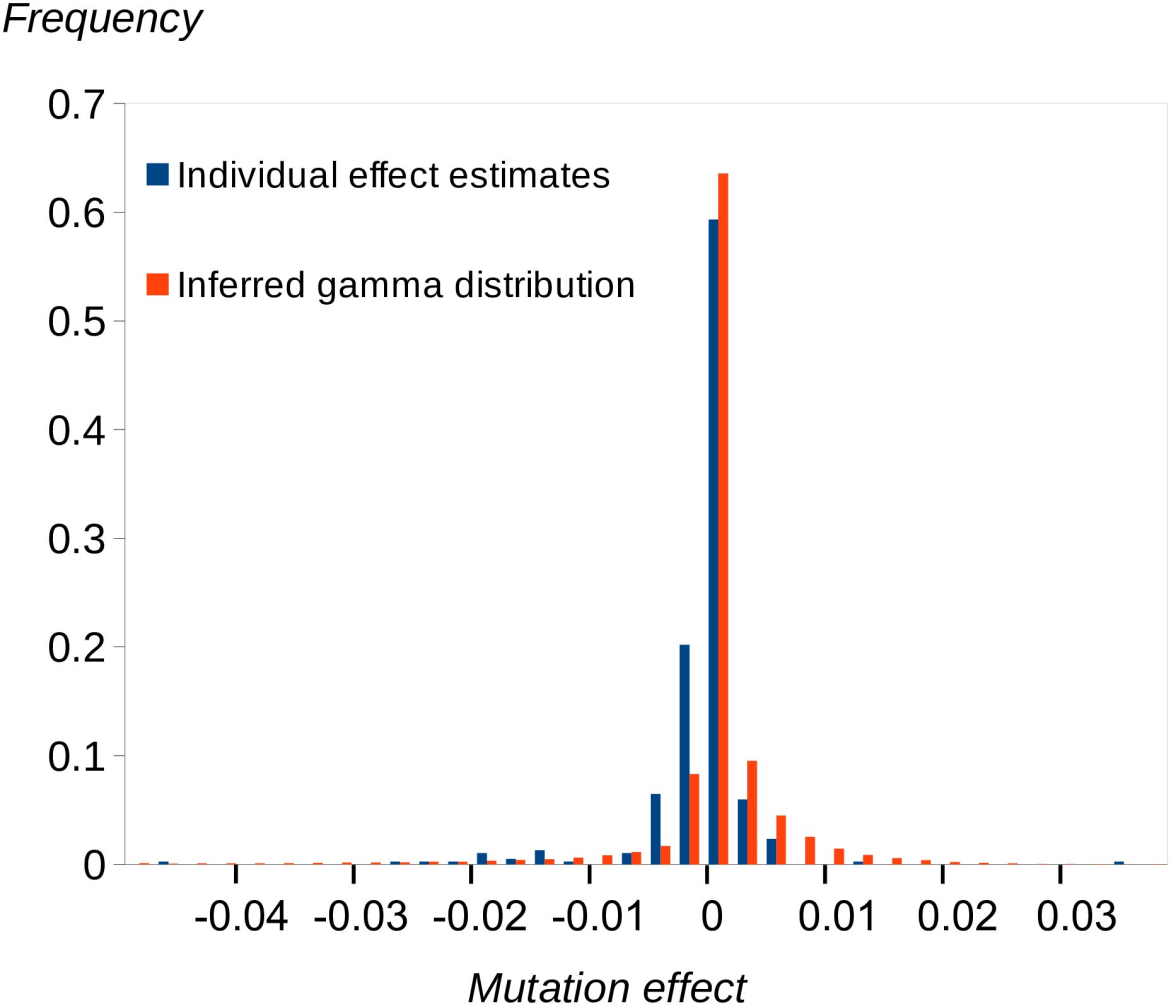
The estimated reflected gamma distribution of effects (inferred gamma distribution) compared to the distribution of posterior mean estimates for the effects of the individual mutations (individual estimates).

**Fig 4:**
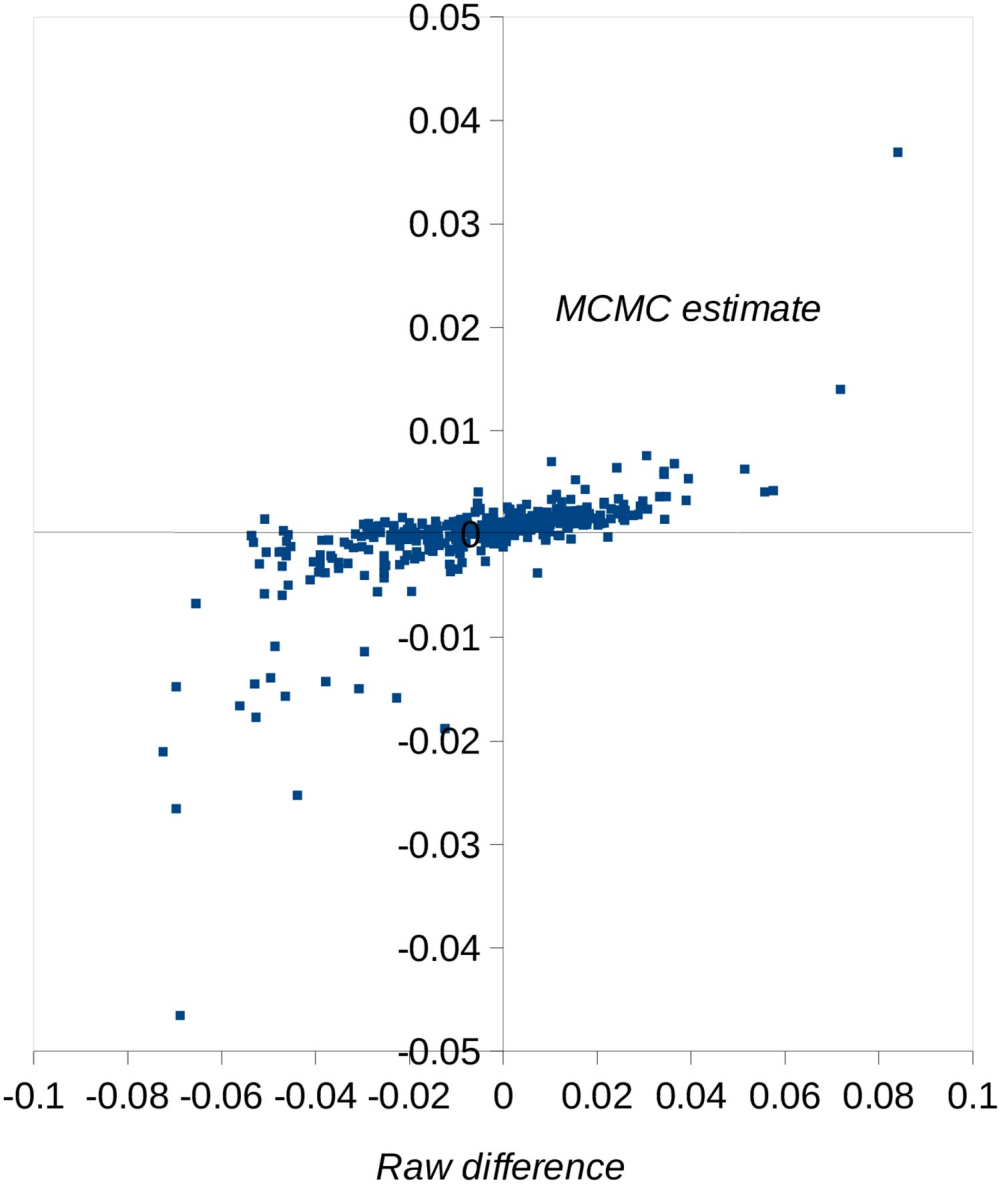
Relationship between estimated fitness effects of mutations obtained by MCMC and estimates obtained from the difference in mean growth rate between recombinant lines carrying the mutant and wild-type allele (raw difference). Raw difference estimates were calculated within MA line genotypes, excluding the ancestral lines (which are homozygous mutant or wild type for all mutations carried by a MA line). MA, mutation accumulation; MCMC, Markov chain Monte Carlo.

We first investigated models in which mutation effects fall into discrete categories, including a zero-effect category. Under a two-category model, there is a strong signal of growth rate–reducing mutations (estimated effect ≈ −3%), consistent with the overall negative effect of spontaneous mutations we previously observed. The majority of mutations (approximately 96%) are, however, allocated to the zero-effect class. Under a three-class model, most mutations are also allocated to the zero-effect class, there is a negative-effect category with similar fitness effect and frequency as in the two class model, and a third category of positive-effect mutations (effect ≈ +2% on fitness). The frequency of positive-effect mutations is approximately 7%, but the credible interval is very wide. We then analysed a two-sided gamma distribution model, in which there are different means for the distributions of positive- and negative-effect mutations. Arguably, this is more realistic than the multicategory model, which assumes that mutation effects are invariant within a category. Consistent with the results from the analysis of the model with three discrete categories, there are both negative- and positive-effect mutations, and the proportion of positive-effect mutations is surprisingly high (about 80%). The distributions for negative- and positive-effect mutations are highly leptokurtic (i.e., the estimate of the shape parameter is approximately 0.3), and the absolute means of the distributions are both <1%, reflecting the concentration of density around zero. It appears that the effects of positive mutations are smaller than those of negative mutations, and the amount of mutational variance contributed by positive-effect mutations is about 20% that of negative-effect mutations. The estimated two-sided gamma distribution of effects is compared to the frequency distribution of the estimated effects of the individual mutations in Fig 3. Overall, the fit to the gamma distribution is reasonable. There is one mutation with a positive effect of +5% (a G→C mutation in the 3′UTR of a gene on Chromosome 6 of unknown function) and several mutations with absolute negative or positive effects > 1%. The annotations associated with the 10 mutations with the highest absolute effects (i.e., the most extreme 2.5%) are shown in S6 Table. There is no significant enrichment of any annotation we tested for these most extreme effects (or for the most extreme 5%). A plot of the estimated fitness effects of the mutations obtained by MCMC and estimates obtained simply as the difference in mean growth rates between RLs carrying the mutant or wild-type allele (Fig 4) shows that these estimates are strongly positively correlated. However, mutant effects estimated under MCMC tend to be shrunk towards zero, particularly if they are close to zero, as expected under a random-effects model.

Why do we infer the presence of a high proportion of positive-effect mutations? This result does not align well with the data: taking an absolute estimated fitness effect of 0.01 as a threshold, there are about six times more negative- than positive-effect mutations exceeding this value (Fig 4). This is reflected in an approximately 4-fold-smaller estimate of the mean absolute effect for positive- than negative-effect mutations (assuming a two-sided gamma DFE, Table 3). This difference in mean between the two sides of the DFE will presumably then result in many mutations with effects close to zero spending more time in the MCMC chain in the positive-effect state, thereby inflating the proportion of positive-effect mutations. There are, however, several biological explanations for a high proportion of positive-effect mutations [27]. One possibility is that mutations that increase fitness are common in natural populations, and this is reflected in MA experiments [15,28]. An alternative view is that deleterious mutations predominate in nature, principally because organisms are well adapted to the environments they typically experience [29]. Consistent with this, functional elements of the genome are typically conserved [30], and analysis of the frequency of amino acid and synonymous polymorphisms within populations suggests that advantageous amino acid mutations are infrequent [31]. A second possible explanation is that the algae were assayed in an environment that the species does not encounter in the wild, and some mutations that are deleterious in nature increase growth rate in the laboratory. A third possibility is that natural selection could not be prevented during the MA experiment, and there was either positive selection for mutations increasing growth or negative selection acting on mutations decreasing growth rate. This could take the form of between colony selection, if the fastest-growing colonies were picked preferentially. Alternatively, there could be within-colony selection, if new advantageous mutations occurring during colony expansion rise in frequency, or new deleterious mutations are removed during colony expansion. The effective population size was approximately 7 [20], and we infer that few mutations have positive effects > 10%, so any substantial selection for positive-effect mutations seems unlikely. On the other hand, deleterious mutations with effects > 10% (including lethal or near-lethal mutations) would be underrepresented.

The inferred DFE is highly leptokurtic, implying that many mutations have a very small effect (we do not know whether their effects are positive or negative), and there is a long tail of large-effect mutations (which are mainly of negative effect). Under the reflected gamma distribution model, the shape parameter of the distribution of negative- and positive-effect mutations is approximately 0.3. This is close to the value assumed by Kimura [2,3] when analysing the nearly neutral model of molecular evolution. Given that a high proportion of sites in the *Chlamydomonas* genome are in protein-coding exons, our inferred DFE is therefore consistent with the observation that amino acid variation is relatively insensitive to *N*_*e*_ [1,3]. Inferring the detailed shape of the DFE for mutations with very small effects is, however, limited by experimental resolutions. Our inferred DFE is also relevant to the narrow range of variation observed at synonymous and noncoding sites [32], if such sites become effectively selected in populations of large effective size. Such a leptokurtic distribution also has implications for the response to artificial selection and maintenance of variation for quantitative traits. If mutation effects are drawn from a leptokurtic distribution, then the response from new mutations builds up quicker than under the infinitesimal model but is more variable, since response depends on the chance appearance and fixation of mutations with large effects [5]. A weak relationship between genetic variance for fitness or a correlated trait and *N*_*e*_ is also predicted [6].

To our knowledge, our approach of crossing MA lines to their ancestors and genotyping and phenotyping the crosses has not been previously attempted. It is related to that applied to induced mutations in RNA viruses [33] and in mismatch repair–deficient *Escherichia coli* [34]. A limitation of our approach is that some mutations we previously identified by whole-genome sequencing [22] were not amenable to genotyping, and therefore, parameter estimates for those mutations we could genotype will be biased to some extent. Specifically, some classes of mutations, such as large indel events or transposable element insertions, were not detectable by our short-read sequencing study or may have occurred in regions that could not be aligned to the reference genome [22]. The approach is limited by the precision of phenotyping for mutations with small effects on growth rate. In general, laboratory-based measurements of mutation effects on fitness have been limited to those stronger than 10^−3^ [35]. Mutations with effects of this magnitude or below will be allocated to the zero-effect category under the discrete class model or will have estimated effects close to zero under the two-sided gamma distribution model. Such mutations might be effectively selected in natural populations, however. The approach therefore has the capability of informing about mutations that may be under such strong selection in nature and, as such, rarely segregate in natural populations. Other approaches that focus on the frequency distribution of segregating polymorphisms [9–12] inform about weakly selected mutations and therefore complement the present approach.

## Materials and methods

### MA lines and the ancestral strain

Production and sequencing of MA lines of six strains of *C*. *reinhardtii* have been described previously [20,22]. Here, we focus on MA lines derived from CC-2931, a strain first sampled in Durham, North Carolina, United States, in 1991 that has a typical mutation rate among several strains we investigated [22] and decreasing mean fitness with increasing mutation number [19].

The MA lines and their ancestral strain are of the same mating type (mt−), so we first produced a ‘compatible ancestor’ to which the MA lines could be crossed. This was done by backcrossing CC-2931 to a strain of the opposite mating type (CC-2344, mt+) for 13 generations with the aim of producing a strain identical to CC-2931, except for the region around the mating type locus on Chromosome 6. Genome sequencing of the compatible ancestor (using the method described in [12]) unexpectedly revealed, however, non-CC-2931 regions not only on Chromosome 6 but also on Chromosomes 4, 5, and 16 (S9 and S10 Figs), constituting a total of 7.6% of the genome and leaving 13 pure CC-2931 chromosomes. We dealt with this issue by including markers for these regions as factors in the analyses (see Inference of the distribution of effects of mutations for growth rate).

### Generation of first-generation RLs

For each MA line, we set up nine independent matings with the compatible ancestor and collected 32 RLs from each to obtain a total of 288 RLs per MA line. Matings were set up by inoculating cultures for both parents into 200 μl of liquid Bold’s medium [36] and incubating these under standard growth conditions (23 °C, 60% humidity, constant white light illumination) while shaking at 180 rpm for 4 days. Nitrogen-free conditions are required to trigger mating in *C*. *reinhardtii* [37], so we centrifuged the cultures (3,500*g*, 5 minutes), removed the supernatant, and added 200 μl of nitrogen-free liquid Bold’s medium. We then mixed 50 μl each of MA line and compatible ancestor cultures and incubated the matings for approximately 24 hours under standard growth conditions to allow zygotes to form at the surface. The zygote mats were transferred to petri dishes containing Bold’s agar and incubated in the dark for 5 days to allow zygote maturation. To kill any vegetative cells associated with the zygote mats, the petri dishes were exposed to chloroform for 45–60 seconds. Subsequently, the petri dishes were incubated under standard growth conditions until the matured zygotes had germinated. As controls for the chloroform treatment, 30 μl of both of the unmated parents of each of the mating reactions were subjected to the same procedure, and the respective mating reaction was discarded if any growth was observed. After successful germination, 2 ml of liquid Bold’s medium was added to the petri dishes to allow the germinated cells to go into suspension. The suspensions were then diluted and spread onto new petri dishes containing Bold’s agar and incubated under standard growth conditions until individual colonies had grown sufficiently to be picked. Initially, 36 individual clones representing individual RLs were picked from each mating and transferred into 200 μl liquid Bold’s medium and incubated under standard growth conditions while shaking at 180 rpm for 3 days. Finally, 32 of the 36 picked RLs were transferred onto Bold’s agar in 7-ml bijou containers for long-term storage.

### Sample preparation for DNA extraction and genotyping

We used the competitive allele-specific PCR (KASP, Kompetitive Allele Specific PCR) technology to genotype the RLs of each MA line, the corresponding MA line, the compatible ancestor, and the original unmutated ancestral strain (CC-2931) at the locations of the mutations previously reported for the MA lines [22]. For allele-specific primer design, DNA regions of 1,000 base pairs (bp) surrounding each mutation were extracted from the *C*. *reinhardtii* reference genome (strain CC-503; version 5.3; [38]). The regions were then corrected to match the consensus sequence of the CC-2931 MA lines.

For DNA extraction, we obtained cell pellets of at least 50 mg as follows. We inoculated the RLs and ancestors into 200 μl of liquid Bold’s medium and incubated these under standard conditions with shaking at 180 rpm for 4 days. The cultures were then transferred to individual wells of 6-well plates filled with 6 ml of Bold’s agar and incubated under standard conditions until a thick lawn had grown. Cells were then scraped off, transferred to 2-ml tubes, and frozen at −70 °C. DNA samples were extracted from the frozen cell pellets and genotyped by LGC Genomics (http://www.lgcgenomics.com) using the sequences flanking each mutation of interest.

In addition to genotyping the known mutations, we genotyped markers that distinguish the mating types and the non-CC-2931 regions (S9 Fig). For the mating type locus, we designed markers matching loci specific to the two mating types, the *fus1* locus for the mt+ mating type and the *mid* locus for the mt− mating type. For the non-CC-2931 regions, we included markers for sites that differed between the two strains within these regions.

### Determination of RL mating types by crossing

In addition to using genetic markers, we determined mating type using crosses. In separate mating reactions, we mated each RL with the ancestral strain and with the compatible ancestor, using a modification of the mating protocol described above, in which we extended the incubation period for the mating reaction under standard growth conditions to approximately 48 hours and then incubated plates in the dark for 5 days. To kill vegetative cells, we then incubated the plates for 5 hours at −20 °C, added 100 μl of a Bold’s medium containing twice the amount of nitrogen as Bold’s medium, and incubated the plates under standard growth conditions while shaking at 180 rpm until zygotes had germinated. We assigned mating type for each RL based on the combined results of the mating test and the mating type genotyping test. If one test failed, we used the result of the other. If the tests disagreed or both failed, a mating type was deemed not assignable and was recorded as missing data.

### Measurement of growth rate

To generate growth curves for the individual RLs and their parents (i.e., the corresponding CC-2931 MA line and the compatible ancestor), we inoculated each of these separately into individual wells of 96-well plates containing 200 μl of liquid Bold’s medium. Each plate contained samples from 58 RLs, all derived from the same MA line and their parental lines. We allocated lines randomly among the 60 central wells to avoid plate-edge effects [20] and filled the outer wells with 200 μl of medium to maintain humidity and reduce evaporation in the central wells. All plates were initially incubated for 4 days under standard conditions. On day 4, we transferred 2 μl of each culture to the corresponding well on a new 96-well plate filled with 198 μl liquid Bold’s medium to start the growth assay. As an estimate of cell density, we measured absorbance at 650 nm every 12 hours over a period of 96 hours. We repeated this complete procedure twice in order to have two temporally independent replicates for each RL.

Maximum growth rate can be estimated from each growth curve as the slope of the linear regression of the natural log (ln) of absorbance on time during the exponential phase of growth. Unfortunately, the start and duration of exponential growth varied between growth curves, so we were unable to simply estimate growth over the same time window for each growth curve. Instead, we used the following procedure. For each growth curve, we generated a number of 48-hour time windows that spanned five measurements in our growth curves. The first started at 12 hours and ran to 60 hours, the second started at 24 hours and ran to 60 hours, and so on, until we had all possible windows up until 96 hours. For each window, we then carried out a regression of ln absorbance on time. The slope of this regression line gives us an estimate of the rate of increase during this time period, while the proportion of the total variation in growth rate explained by the linear regression on time (the *R*^2^ value) gives an estimate of how well the linear relationship fits the data. We carried out this procedure for windows of 60 hours (6 time points), 72 hours (7 time points), and 84 hours (8 time points). We then excluded any windows for which the fit of the linear model was not adequate (*R*^2^ < 0.75). We then examined the slope estimates from each of the remaining windows and used the highest estimate as our measure of maximum growth rate for that growth curve. Visual inspection of the fitted lines on the time series showed that this procedure was effective in identifying the period of maximum growth for the variety of observed growth trajectories. For a total of 8 RL replicate time series measures, an adequate fit was not achieved for any of the time windows because of extremely unusual growth trajectories, and these were excluded from further analysis (S1 Table).

### Data processing and preparation

Mutations that were invariant across all samples were considered as artifactual and excluded. We also excluded mutations that were in complete linkage with either the mating type locus or one or more marker from the non-CC-2931 regions (S2 Table). In the case of 21 mutations, only one of the two allele-specific primers worked successfully, and consequently no genotype information on the mutation was available for about 50% of the RLs. We corrected such mutations by changing the missing genotype to the nonamplified allele (S7 Table). We excluded RLs for which genotypes of more than 10% of mutations were missing and/or for which more than 5% of mutations were assigned as heterozygous. *C*. *reinhardtii* is haploid, and multiple heterozygous calls suggest that the RL contains several different genotypes and potentially cross-contamination. The rationale for setting these thresholds for missing data and heterozygous calls came from plotting the distributions of percentages of missing data and heterozygous calls for the complete data set. Only a small number of RLs have more than 10% of missing mutations and/or have more than 5% of the mutations assigned as heterozygous (S11 Fig).

After carrying out the above filtering steps, several missing genotypes remained, so we imputed as many as possible to facilitate analyses. We first assigned missing genotypes for cases in which RLs of apparently identical haplotype originated from the same mating reaction. In a second step, we computed the squared measure of linkage disequilibrium between pairs of mutations (*r*^2^) [39]. We then examined the remaining mutations that have missing genotypes in turn. If *r*^2^ between a mutation and its neighbouring mutation was above 0.7, we imputed its allelic state using the state of the neighbouring mutation. Of the total 205,351 data points across all MA lines, 1,982 (0.97%) were initially missing (= number of mutations × number of samples including all replicates of RLs and ancestors). With our imputation approach, we were able to impute 1,766 (89%) of them so that only 216 (0.11%) missing data points remained.

### Relationship between number of mutations and growth rate

To examine the relationship between RL growth rate and the number of mutations carried, we fitted a linear mixed model to the combined data set from all 6 MA lines and to the individual MA lines, with growth rate as the response variable and the number of mutations carried as a continuous linear predictor. To control for other sources of variation, we also fitted mating type and all markers for the non-CC-2931 regions as fixed factors and MA line, haplotype, and growth assay plate as random factors. The significance of the number of mutations was examined by comparing models with and without this term, using a likelihood ratio test. The analysis was also done for specific mutation types (SNP, indel, exonic, intronic, intergenic). Models were fitted using the *lme4* [40] package in R [41]. The data are provided in S2 Data, and the R code is provided in S3 Data.

### Inference of the distribution of effects of mutations for growth rate

We developed an MCMC approach (S4 Data) to infer the distribution of effects of mutations for growth rate, assuming two kinds of models. We modelled a discrete distribution in which each mutation’s effect fell into one of a number (*n*_*c*_) of categories and a two-sided gamma distribution allowing different parameters for the distributions of negative- and positive-effect mutations. To control for the effects of mating type and presence/absence of non-CC-2931 chromosomal regions, we included *n*_*f*_ two-level fixed effects. Normally distributed plate effects and residual effects and the overall mean were also fitted. When analysing a merged data set of all six MA line crosses (S5 Data), a different mean was fitted for each MA line, and any RL with one or more missing genotypes was excluded.

The data for the RLs bred from one MA line are represented in Table 6. Let *n*_*b*_ be the number of RL observations, and let *n*_*m*_ be the total number of mutations in the MA line. Mutations are encoded in a *n*_*b*_ × *n*_*m*_ matrix, **M**, whose elements (0 or 1) indicate the presence or absence of a mutation in an RL. The fixed effects associated with each observation are encoded in a matrix, **F**, of dimension *n*_*b*_ × *n*_*f*_ with elements 0 or 1. Plate numbers (*n*_*p*_ levels) and phenotypic values associated with each observation are vectors **r** and **y**, respectively, both of dimension *n*_*b*_.

**Table 6:** Representation of the data from a MA line crossing experiment.

Description	Symbol	Dimension
Number of observations	*n*_*b*_	Scalar
Overall number of mutations	*n*_*m*_	Scalar
Mutation matrix	**M**	*n*_*b*_ × *n*_*m*_
Number of fixed effects	*n*_*f*_	Scalar
Fixed effects matrix	**F**	*n*_*b*_ × *n*_*f*_
Number of plates	*n*_*p*_	Scalar
Plate number vector	**r**	*n*_*b*_
Phenotypic value vector	**y**	*n*_*b*_

For each MCMC iteration, the state of one of the model’s variables (which are elements of vectors or scalars defined in Tables 7–9) is proposed and then accepted or rejected based on change in log likelihood. Posterior distributions of the model variables provide Bayesian parameter estimates.

**Table 7:** Variables in the MCMC model specific to the multicategory model.

Description of variable	Symbol	Dimension	Constraints on value
Vector of mutation categories	**m**	1‥*n*_*m*_	Integer, 0‥ 1 –*n*_*c*_
Vector of mutation effects	**e**	0‥ *n*_*c*_− 1	–
Vector of mutation frequencies	**q**	0‥ *n*_*c*_− 1	$\sum_{1}^{n_{c}-1}q$ < 1

Abbreviation: MCMC, Markov chain Monte Carlo.

**Table 8:** Variables of the model common to the multicategory and two-sided gamma distribution models.

Description of variable	Symbol	Dimension	Constraints on value
Vector of fixed effects	**f**	1‥ *n*_*f*_	–
Vector of plate effects	**p**	1‥ *n*_*p*_	–
Random plate effect variance	*V*_*p*_	Scalar	–
Overall mean	*ȳ*	Scalar	–
Residual variance	*V*_*e*_	Scalar	–

**Table 9:** Variables in the model specific to the two-sided gamma distribution model.

Description of variable	Symbol	Dimension	Constraints on value
Vector of mutation sign indicators	**μ**	1‥*n*_*m*_	Integer, 0‥1
Matrix of mutation effects	**E**	[0‥1] × [1‥*n*_*m*_]	Positive real number
Vector of frequencies of negative and positive-effect mutations	**q**	0‥1	0 < *q*_0_ < 1
Vector of shape parameters	**α**	0‥1	Positive real number
Vector of scale parameters	**β**	0‥1	Positive real number

### Multicategory model

Under this model, one category of mutations has no effect on fitness, and the remaining categories have nonzero effects. The mutation category vector (**m**) specifies the category in which each mutation currently resides, the value zero signifying that a mutation is in the zero-effect category (Tables 7 and 8). Elements of **m** are proposed by randomly picking an integer in the range 0‥1 –*n*_*c*_, which is different from the current value. State variables for the effects and frequencies associated with each category are encoded in vectors **e** and **q**, respectively. The first element (*e*_0_) of **e** is fixed at zero, since it is the effect of the invariant zero-effect class, and the first element of **q** is set to $q_{0}=1-\sum_{i=1}^{n_{c}-1}q_{i},$ the frequency of the zero-effect class. Proposals for the remaining elements of **q** are random uniform deviates added to the current value. Changes to the values of all other variables are drawn from normal distributions with mean zero. The variances of the uniform and normal distributions of proposal deviates are adjusted during a burn-in phase so that about 25% of proposals are accepted.

Proposals are accepted or rejected by applying the Metropolis-Hastings algorithm based on the log likelihood of the data and the priors (which are designed to be uninformative), given the parameter values. The overall log likelihood contains contributions from the numbers of mutations in different categories, their frequencies, the random plate effect, and each observation, which are considered independent. Let **v** be a vector of dimension 0 to 1 –*n*_*c*_ containing the numbers of mutations in each of the *n*_*c*_ categories in the current proposal, and *multinomial*(*n*_*c*_, **q**, **v**) be the probability of sampling **v** from a multinomial distribution parameter **q**. Let *normal*(*y*, *ȳ*, *V*_*e*_) be the normal distribution probability density function for point *y* with mean *ȳ* and variance *V*_*e*_. Let *g*_*i*_ be the genotypic value of RL *i*, which is the sum of the effects of the mutations it carries. This is calculated from the set of mutations carried by the RL (specified in **M**), the categories into which these mutations fall (specified in **m**), and the effect associated with each category (specified in **e**):

$$g_{i}=\sum_{j=1}^{n_{m}}e[M_{ij}\times m_{j}],$$ (1)

where the square brackets denote vector or matrix indexing, i.e., *e*[*x*] = *e*_x_. The overall log likelihood of the data is then:

$$\begin{array}{c} logL=\sum_{i=1}^{n_{m}}log(q[m_{i}])+log(multinomial(n_{c},\text{q},\text{v}))+\sum_{i=1}^{n_{p}}log(normal(p_{i},0,V_{p}))+\sum_{i=1}^{n_{b}}log(normal(y_{i}-g_{i}-\sum_{j=1}^{n_{f}}F_{ij}\times f_{j}-p[r_{i}],ȳ,V_{e})) \end{array}$$ (2)

Note that the model with three categories (including a zero-effect category) is equivalent to a model with a mixture of two gamma distributions both with shape parameters → ∞ plus a zero-effect category (see below).

### Two-sided gamma distribution model

Under the two-sided gamma distribution model (whose variables are defined in Tables 7 and 9), the current state of a mutation in the MCMC is defined by two variables. The first is whether the mutation has a negative or positive effect, encoded as 0 or 1 in vector **μ**. The second variable is the genotypic effect of the mutation, encoded in a matrix **E** of dimension [0‥1] × [1‥*n*_*m*_]. The current value of the element of **μ** selects the mutation’s current genotypic effect; i.e., for mutation *i*, the genotypic value is *E*[*μ*_*i*_][*i*]. The frequencies of negative- and positive-effect mutations are encoded in vector **q**. The scale and shape of the gamma distributions for negative- and positive-effect mutations are encoded in vectors **α** and **β**, respectively. Proposals for *q*_0_ are random uniform deviates added to the current value, such that 0 < *q*_0_ < 1 and *q*_1_ = 1 − *q*_0_. A proposal for an element of **μ** is 0 if the current value is 1 and vice versa. Changes to the values of all other variables are drawn from normal distributions with mean zero with adjustment during the burn-in as described above.

Let gamma(*x*, *α*, *β*) be the gamma distribution PDF for point *x*, given scale and shape parameters *α* and *β*, respectively. Let **v** be a vector (with two elements indexed by 0 and 1) containing the numbers of mutations with negative and positive effects in the current proposal, and *binomial*(*q*_0_, *v*_0_) be the probability of sampling *v*_0_ negative-effect mutations, given that the frequency of negative-effect mutations is *q*_0_. Let *g*_*i*_ be the genotypic value of RL *i* (the sum of the effects of the mutations it carries, as above). This is calculated from the set of mutations carried by the line (specified in **M**), the types into which these mutations fall (i.e., negative or positive specified in **μ**), and the effect of each mutation (specified in **E**):

$$g_{i}=\sum_{j=1}^{n_{m}}E_{\mu_{j}j}\times \delta_{j}\times M_{ij},$$ (3)

where *δ*_*j*_ takes the value −1 if *μ*_*j*_ is 0 and +1 if *μ*_*j*_ is 1. The overall log likelihood of the data is:

$$\begin{array}{c} logL=\sum_{i=1}^{n_{m}}\{log(gamma(E_{\mu_{i},i},\alpha [\mu_{i}],\beta [\mu_{i}]))+log(q[\mu_{i}])\}+log(binomial(q_{0,}v_{0}))+\sum_{i=1}^{n_{p}}log\{normal(p_{i},0,V_{p})\}+\sum_{i=1}^{n_{b}}log(normal(y_{i}-g_{i}-\sum_{j=1}^{n_{f}}F_{ij}\times f_{j}-p[r_{i}],ȳ,V_{e})) \end{array}$$ (4)

We considered models in which the shape parameter of the gamma distributions for negative- and positive-effect mutations were the same or allowed to be different.

### Running the MCMC

MCMC runs started with a burn-in of 10^8^ iterations for multicategory models or 10^9^ iterations for two-sided gamma distribution models. Parameter values were then sampled every 10,000 iterations for 9 × 10^8^ iterations for multicategory models or for 5 × 10^9^ iterations for two-sided gamma distribution models. From each sampled iteration, the vector of state variables was stored for generation of plots of parameter values against iteration number or posterior density plots. The mode of the posterior distribution was taken as the parameter estimate and 95% credible intervals computed on the basis of ranked parameter values. Priors for fixed effects, plate effects, and the overall mean and variance were uninformative. The prior for mutation frequencies was a uniform distribution bounded by 0 and 1 and was therefore informative. Priors for the mutation effect parameters (under the multiple category model) were uniform in the range ±0.5 phenotypic standard deviations. Priors for the mean of the gamma distributions (under the two-sided gamma distribution model) were uniform in the range zero to 0.5 phenotypic standard deviations. Priors for the shape parameters of the gamma distributions were uniform in the range 0.1 to 100.

To check whether signals detected in the data were genuine, phenotypic values for fitness were permuted among backcross lines within plates without replacement. The distribution of estimates for parameters of interest obtained from such permuted data sets were computed. Significant estimates from the original data were expected to lie outside these distributions.

Model comparison was carried out using the BIC [42]: $\text{BIC}=k\;\text{log}(n)-2\text{log}(\hat{L})$, where *k* is the number of parameters estimated in the model (in the case of the two-sided gamma distribution models, this number includes twice the number of mutations), *n* is the number of observations and $\hat{L}$ is the maximum likelihood for the model. We used the convention that if BIC(model A)–BIC(model B) < −10, there is strong evidence in favour of model A over model B [43].

### Simulations

To check the method, simulated data sets with either two, three, or four categories of mutational effects or a two-sided gamma DFE and 40 mutations in each data set were analysed as described above, while assuming the same model as simulated (S6 Data). In all cases, posterior modes are close to the parameter values of the simulations (S12–S15 Figs).

## Supporting information

S1 Fig Proportion of ancestral (red), derived (blue), and missing (grey) states at each mutated position for each haplotype. Haplotypes are sorted from left to right according to the proportion of ancestral states at the mutated positions. Underlying data for this figure can be found in S7 Data.
(TIFF)

S2 Fig Results of MCMC analysis of combined data set of RLs from six MA lines, assuming a model with two categories of mutational effects, one of which has an effect of zero. Bayesian posterior density plots are for parameters *e*_1_ and *q*_1_ (the effect and proportion of mutations in category 1, respectively). Software and commands underlying this figure can be found in S4 Data. MA, mutation accumulation; MCMC, Markov chain Monte Carlo; RL, recombinant line.
(TIF)

S3 Fig Values of sampled parameters *e*_1_ and *q*_1_ (effect and frequency of mutations) in MCMC run. The mutation effect is shown unscaled by the trait mean. Software and commands underlying this figure can be found in S4 Data. MCMC, Markov chain Monte Carlo.
(TIF)

S4 Fig Results of MCMC analysis of combined data set of 6 MA line backcrosses assuming a model with three categories of mutational effects (including one category with an effect of zero). Bayesian posterior density plots are shown for *e* and *q* parameters (the effect of and proportion of mutations, respectively, in the two finite-effect categories). Software and commands underlying this figure can be found in S4 Data. MA, mutation accumulation; MCMC, Markov chain Monte Carlo.
(TIF)

S5 Fig Values of sampled parameters *e* and *q* (effect and frequency for negative- [index 1] and positive-effect [index 2] mutations) in MCMC run. The mutation effects are shown unscaled by the trait mean. Software and commands underlying this figure can be found in S4 Data. MCMC, Markov chain Monte Carlo.
(TIF)

S6 Fig Distribution of posterior modal estimates for mutation effect parameters *e*_1_ and *e*_2_ obtained from analysis of data sets in which phenotypic values are permuted within plates with replacement under the three mutation category model. Software and commands underlying this figure can be found in S4 Data.
(TIF)

S7 Fig Values of sampled parameters *q*_1_ (frequency of positive-effect mutations), β (gamma distribution shape parameter), and means for negative- and positive-effect mutations in MCMC run for the two-sided gamma distribution with different means for negative- and positive-effect mutations. The mean mutation effects are shown unscaled by the trait mean. Software and commands underlying this figure can be found in S4 Data. MCMC, Markov chain Monte Carlo.
(TIF)

S8 Fig Values of sampled parameters *q*_1_ (frequency of positive-effect mutations), β (gamma distribution shape parameter), and mean absolute effect of mutations in MCMC run for the two-sided gamma distribution with the same means for negative- and positive-effect mutations. The mean absolute mutation effect is shown unscaled by the trait mean. Software and commands underlying this figure can be found in S4 Data. MCMC, Markov chain Monte Carlo.
(TIF)

S9 Fig SNP densities along Chromosomes 4, 5, 6, and 16 between the compatible ancestor for CC-2931 and its two ancestral strains. SNP densities were calculated for 80-kb windows along the chromosomes between the compatible ancestor and CC-2931 (red) and between the compatible ancestor and the mating type + donor strain (black). A mutation density of 0 indicates no genetic differences between the compatible ancestor and the strain it was compared to. The positions for the markers for the non-CC-2931 regions (blue) and for the mating type marker (green) are indicated. Underlying data for this figure can be found in S8 Data.
(TIFF)

S10 Fig SNP densities along Chromosomes 1–3, 7–15, and 17 between the compatible ancestor for CC-2931 and its two ancestral strains. SNP densities were calculated for 80-kb windows along the chromosomes between the compatible ancestor and CC-2931 (red) and between the compatible ancestor and the mating type + donor strain (black). A mutation density of 0 indicates no genetic differences between the compatible ancestor and the strain it was compared to. Underlying data for this figure can be found in S9 Data.
(TIFF)

S11 Fig Distribution of missing data and heterozygous calls. The distribution of (A) the proportion of missing data, i.e., noncallable mutations across the whole data set, and (B) the proportion of heterozygous calls. Based on these distributions, RLs with >10% missing data and/or >5% heterozygous calls were excluded from all analyses. Underlying data for this figure can be found in S10 Data.
(TIFF)

S12 Fig Posterior density plots for parameters *e*_1_ and *q*_1_ from MCMC analysis of simulated data with two categories of mutational effects, including one zero-effect category. The simulated values were *e*_1_ = 0.25 and *q*_1_ = 0.2. The mutation effects here and in S2 and S3 Figs are expressed in phenotypic standard deviation units. There were 40 mutations simulated and 10,000 observations. Software and commands underlying this figure can be found in S4 Data. MCMC, Markov chain Monte Carlo.
(TIF)

S13 Fig Posterior density plots for *e* and *q* parameters from MCMC analysis of simulated data with three categories of mutational effects, including one zero-effect category. The simulated values were *e*_1_ = −0.3, *q*_1_ = 0.1, *e*_2_ = 0.2, and *q*_2_ = 0.2. Software and commands underlying this figure can be found in S4 Data. MCMC, Markov chain Monte Carlo.
(TIF)

S14 Fig Posterior density plots for *e* and *q* parameters from MCMC analysis of simulated data with four categories of mutational effects, including one zero-effect category. The simulated values were *e*_1_ = 0.4, *q*_1_ = 0.15, *e*_2_ = 0.2, *q*_2_ = 0.2, *e*_3_ = −0.3, and *q*_3_ = 0.1. Software and commands underlying this figure can be found in S4 Data. MCMC, Markov chain Monte Carlo.
(TIF)

S15 Fig Posterior density plots for mean effects of negative (*e−*) and positive mutations (*e+*), gamma distribution shape parameters (*beta−* and *beta+*), and the proportion of positive-effect mutations (*q*_1_) from MCMC analysis of simulated data under a two-sided gamma distribution of mutational effects. The simulated values were *e−* = 0.5, e+ = 0.25, beta− = 0.5, beta+ = 2, *q*_1_ = 0.25. Software and commands underlying this figure can be found in S4 Data. MCMC, Markov chain Monte Carlo.
(TIF)

S1 Table RLs that were excluded from all analyses. RL, recombinant line.
(XLSX)

S2 Table Mutations that were excluded from all analyses. (XLSX)

S3 Table Number of haplotypes from each MA line backcross. MA, mutation accumulation.
(XLSX)

S4 Table Likelihood ratio tests for mixed-model analysis of growth rate as a function of number of different mutation types with 1 degree of freedom. (XLSX)

S5 Table Parameter estimates for the two-mutation-effect category model, including one zero-effect category. (DOC)

S6 Table Effects and mutation types of top 10 absolute effect mutations. (CSV)

S7 Table Mutations that were corrected. (XLSX)

S1 Data Genomic annotations of the mutations. (CSV)

S2 Data Data to examine the relationship between number of mutations and growth rate. (GZ)

S3 Data R code to examine the relationship between number of mutations and growth rate. (TXT)

S4 Data Software and commands to run the MCMC analysis. MCMC, Markov chain Monte Carlo.
(GZ) Click here for additional data file.

S5 Data Data underlying S10 Fig. Merged data set of 6 MA lines with mutation genotypes, fixed effects and growth rate, and input for MCMC analysis. MA, mutation accumulation; MCMC, Markov chain Monte Carlo.
(GZ) Click here for additional data file.

S6 Data Software to simulate data for the MCMC analysis. MCMC, Markov chain Monte Carlo.
(GZ) Click here for additional data file.

S7 Data Data underlying S1 Fig. (GZ)

S8 Data Data underlying S9 Fig. (GZ)

S9 Data Data underlying S10 Fig. (GZ)

S10 Data Data underlying S11 Fig. (GZ)

## Abbreviations

BIC: Bayesian information criterion
DFE: distribution of fitness effects
MA: mutation accumulation
MCMC: Markov chain Monte Carlo
RL: recombinant line
