# Supplementary figures and images for: Inferring the distribution of fitness effects of spontaneous mutations in Chlamydomonas reinhardtii
Source: PLoS Biol. 2019 Jun 26;17(6):e3000192. doi: 10.1371/journal.pbio.3000192 (PMC6615632; doi:10.1371/journal.pbio.3000192)

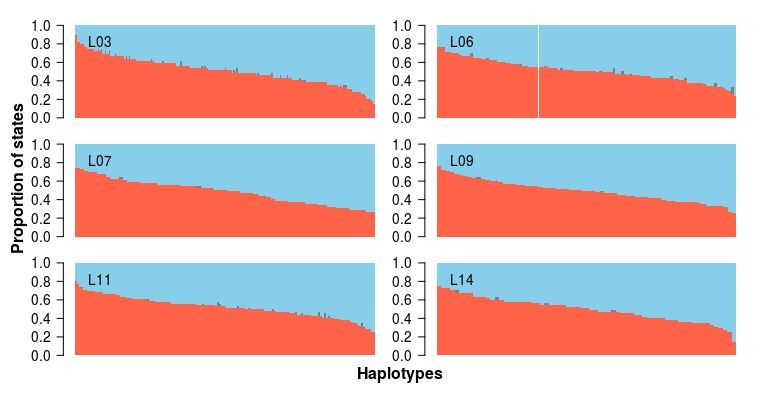

Supplement: S1 Fig — Haplotypes are sorted from left to right according to the proportion of ancestral states at the mutated positions. Underlying data for this figure can be found in S7 Data. (TIFF) [file pbio.3000192.s001.tiff]

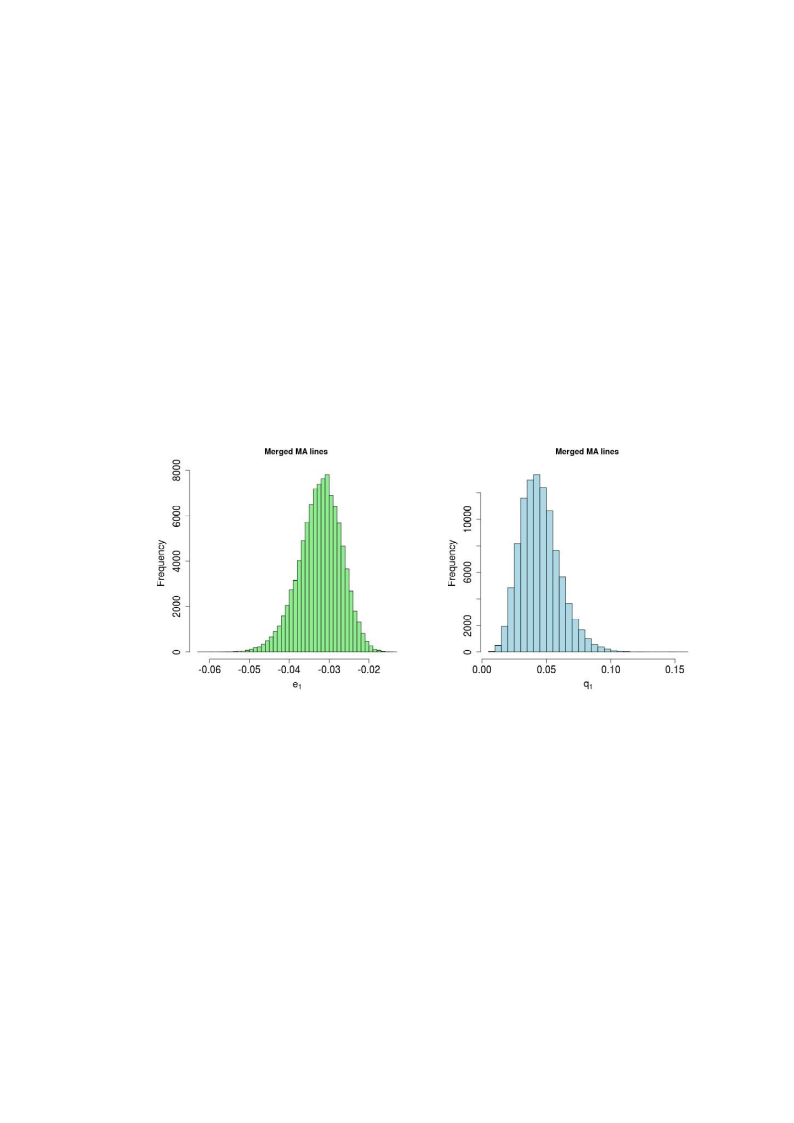

Supplement: S2 Fig — Bayesian posterior density plots are for parameters e1 and q1 (the effect and proportion of mutations in category 1, respectively). Software and commands underlying this figure can be found in S4 Data. MA, mutation accumulation; MCMC, Markov chain Monte Carlo; RL, recombinant line. (TIF) [file pbio.3000192.s002.tif]

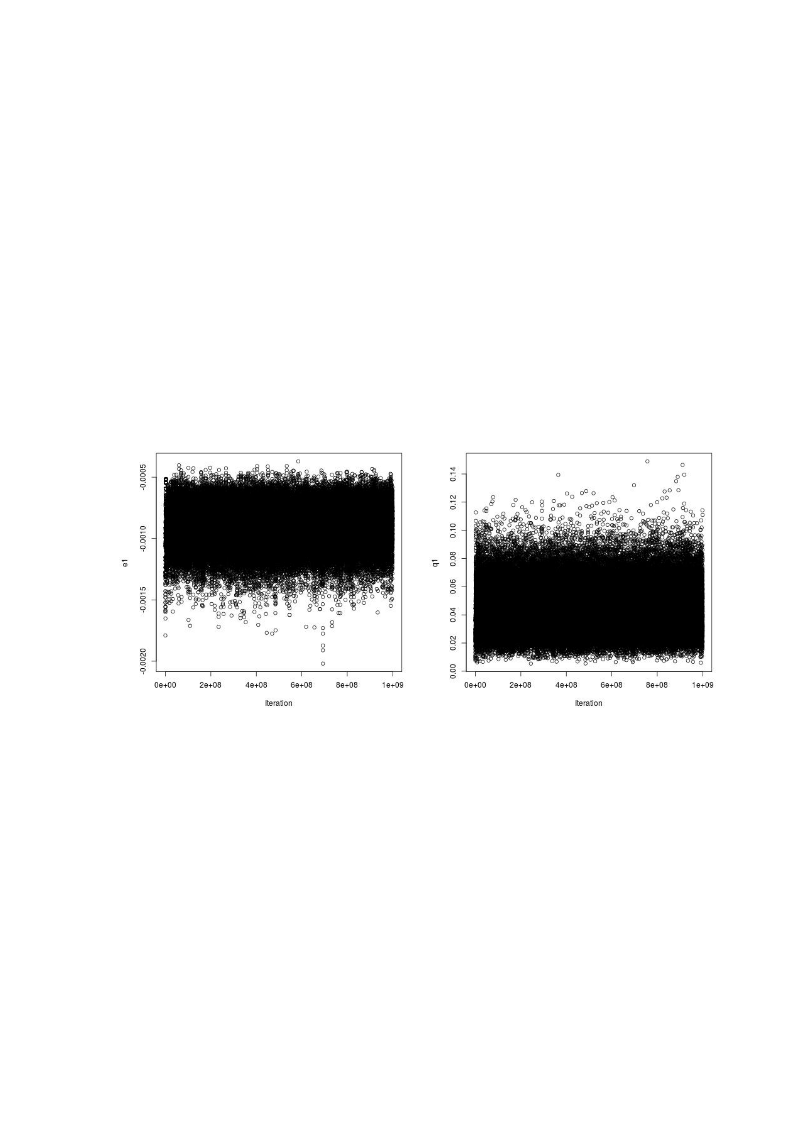

Supplement: S3 Fig — The mutation effect is shown unscaled by the trait mean. Software and commands underlying this figure can be found in S4 Data. MCMC, Markov chain Monte Carlo. (TIF) [file pbio.3000192.s003.tif]

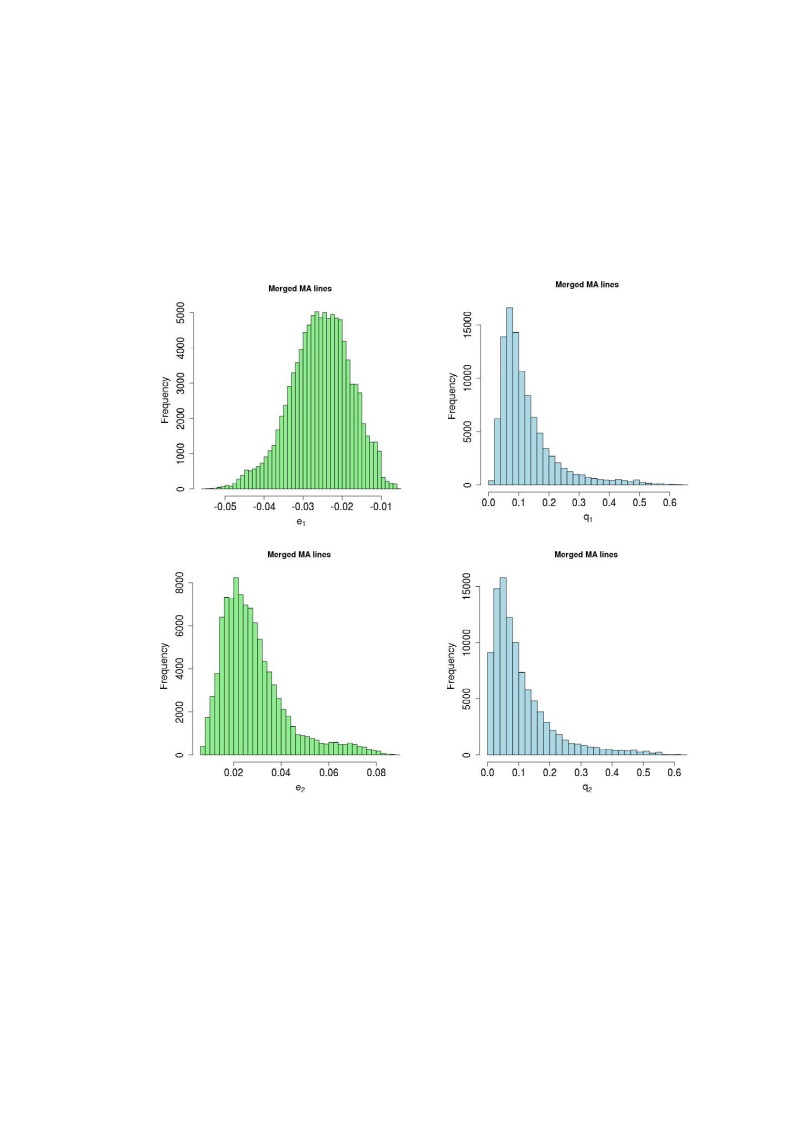

Supplement: S4 Fig — Bayesian posterior density plots are shown for e and q parameters (the effect of and proportion of mutations, respectively, in the two finite-effect categories). Software and commands underlying this figure can be found in S4 Data. MA, mutation accumulation; MCMC, Markov chain Monte Carlo. (TIF) [file pbio.3000192.s004.tif]

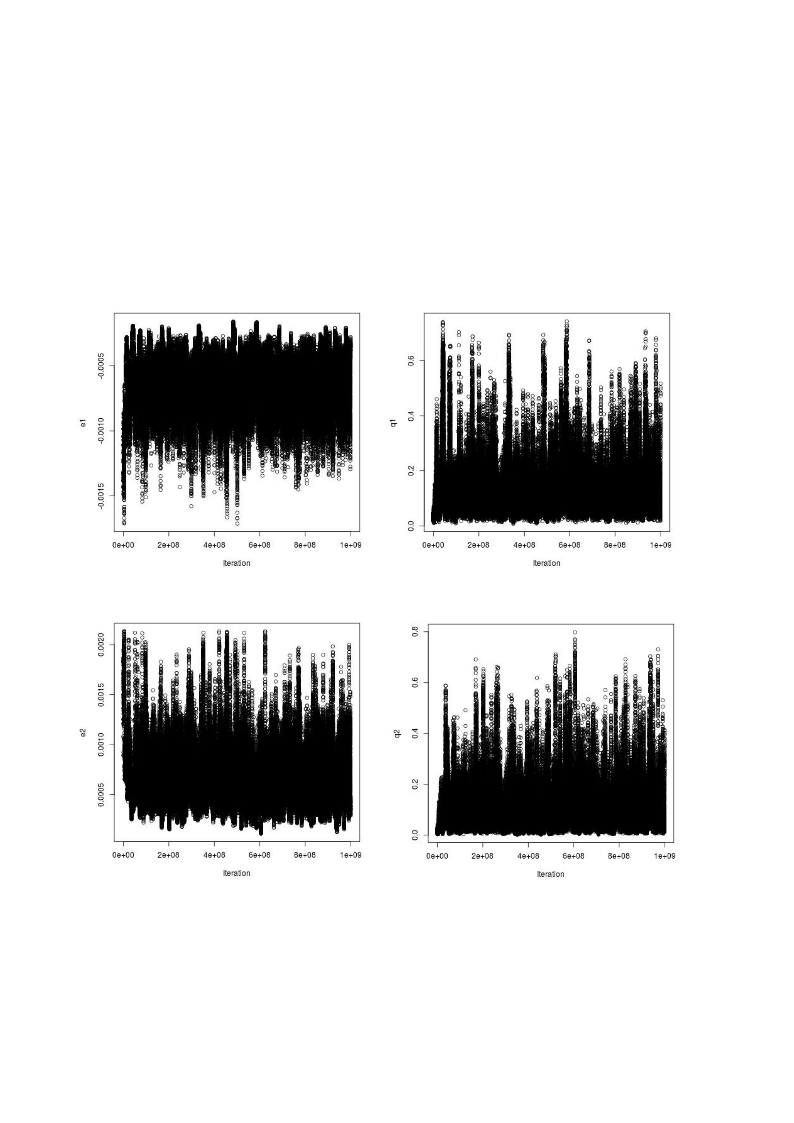

Supplement: S5 Fig — The mutation effects are shown unscaled by the trait mean. Software and commands underlying this figure can be found in S4 Data. MCMC, Markov chain Monte Carlo. (TIF) [file pbio.3000192.s005.tif]

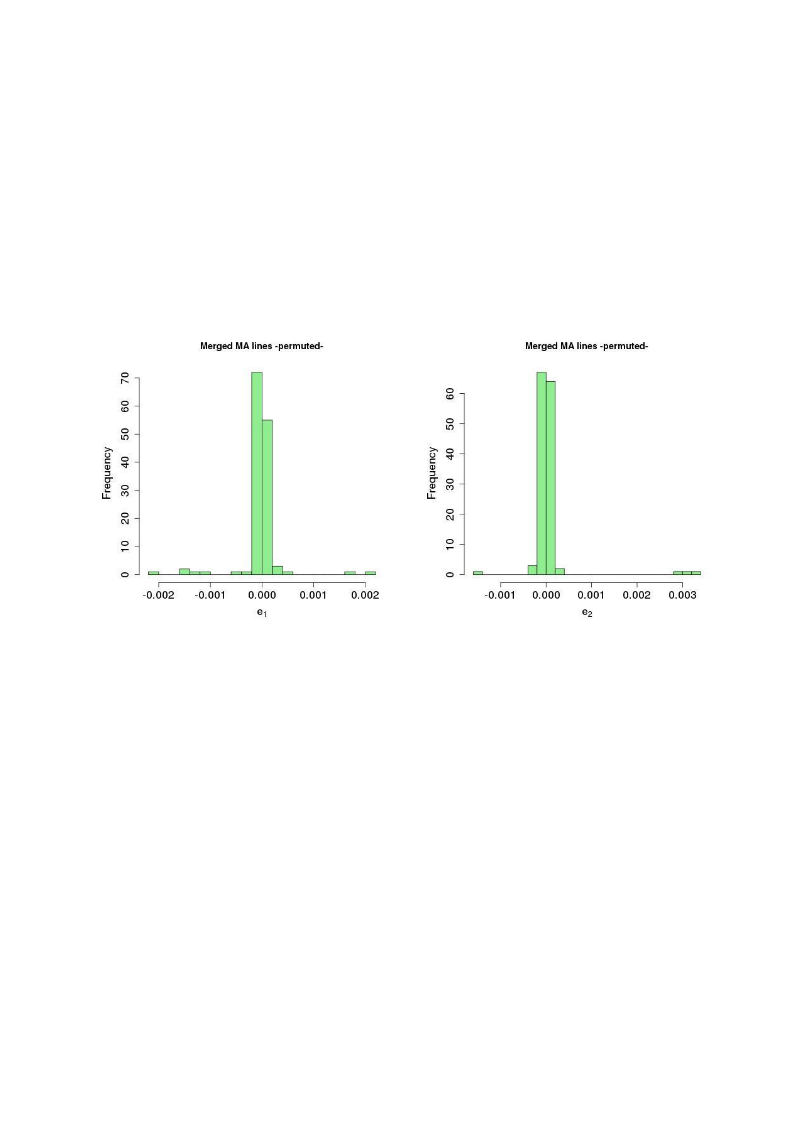

Supplement: S6 Fig — Software and commands underlying this figure can be found in S4 Data. (TIF) [file pbio.3000192.s006.tif]

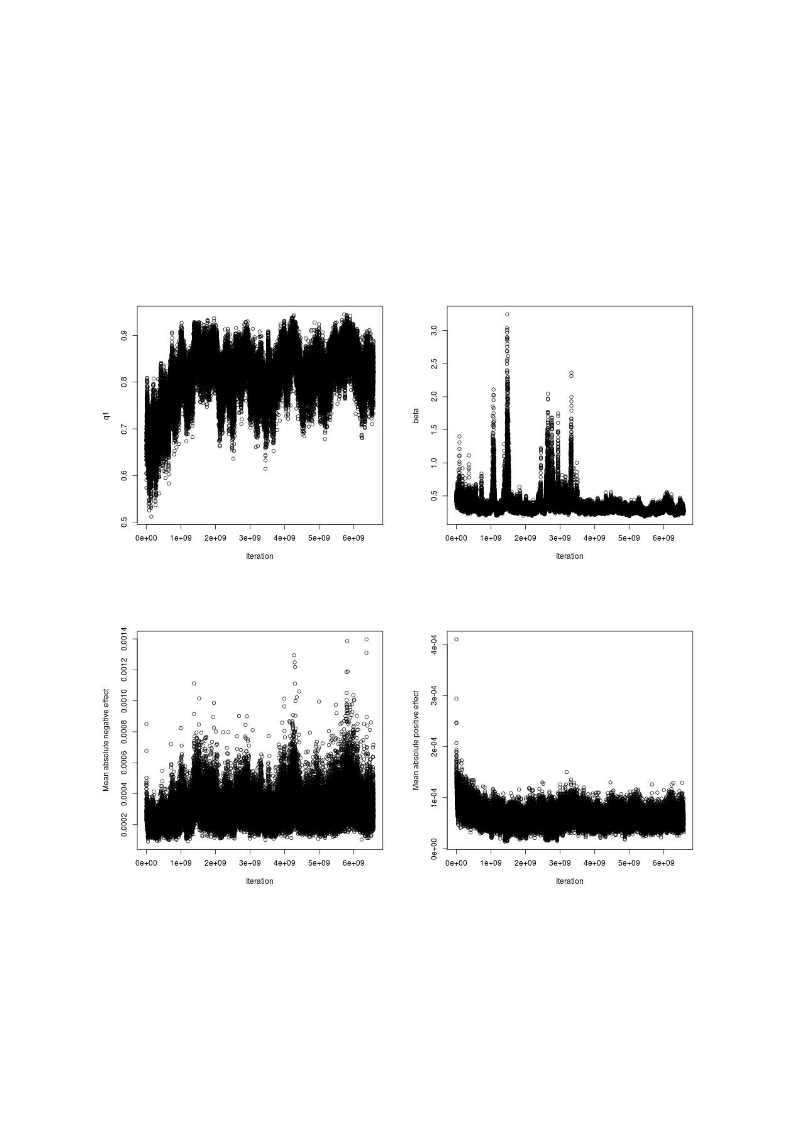

Supplement: S7 Fig — The mean mutation effects are shown unscaled by the trait mean. Software and commands underlying this figure can be found in S4 Data. MCMC, Markov chain Monte Carlo. (TIF) [file pbio.3000192.s007.tif]

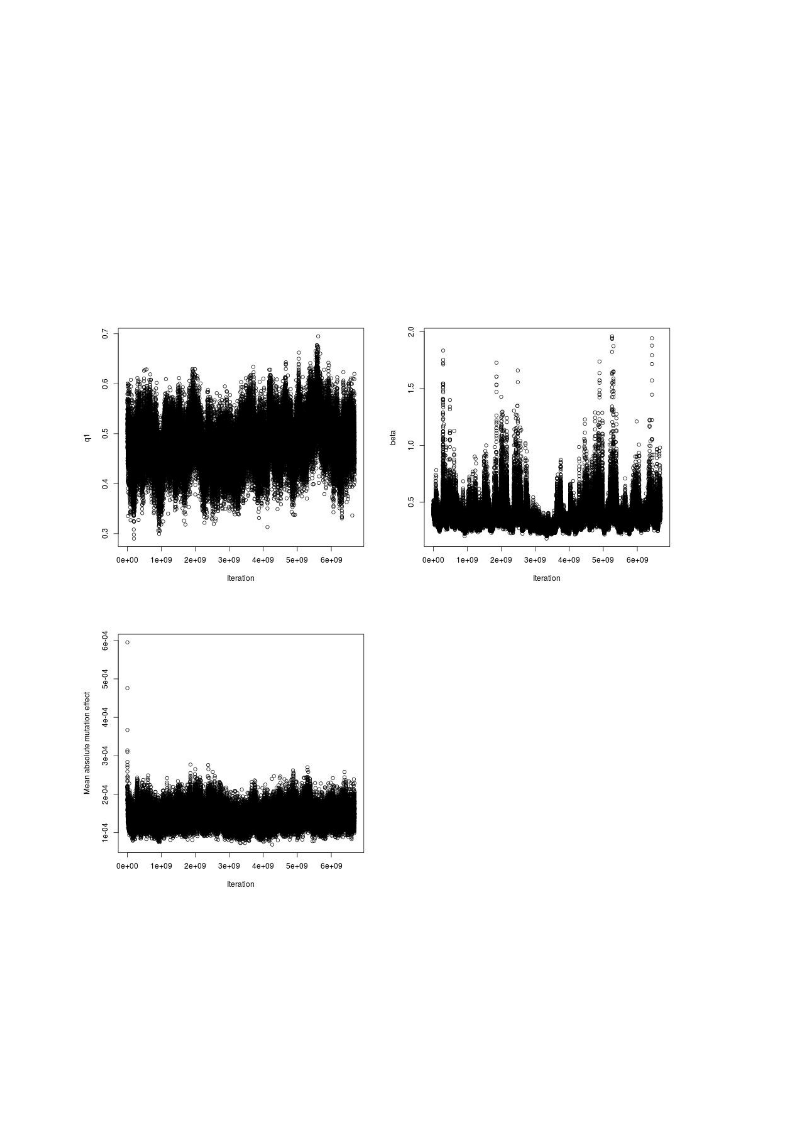

Supplement: S8 Fig — The mean absolute mutation effect is shown unscaled by the trait mean. Software and commands underlying this figure can be found in S4 Data. MCMC, Markov chain Monte Carlo. (TIF) [file pbio.3000192.s008.tif]

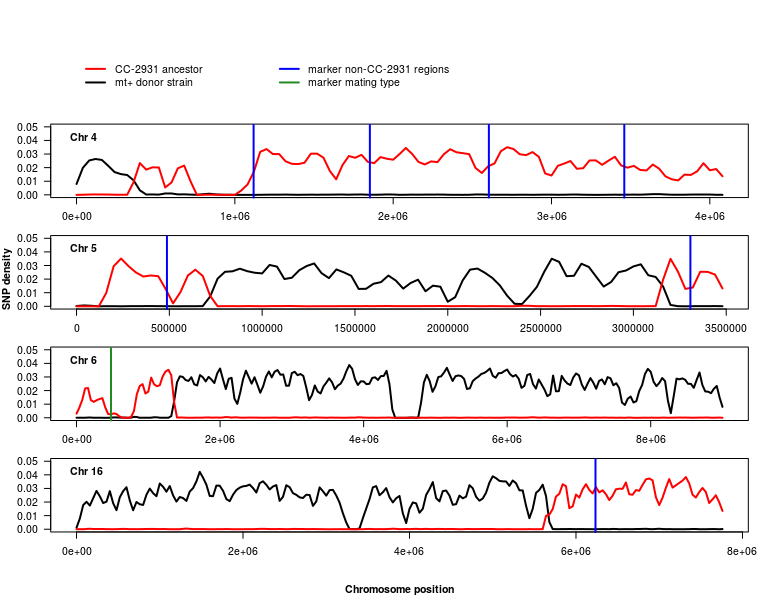

Supplement: S9 Fig — SNP densities were calculated for 80-kb windows along the chromosomes between the compatible ancestor and CC-2931 (red) and between the compatible ancestor and the mating type + donor strain (black). A mutation density of 0 indicates no genetic differences between the compatible ancestor and the strain it was compared to. The positions for the markers for the non-CC-2931 regions (blue) and for the mating type marker (green) are indicated. Underlying data for this figure can be found in S8 Data. (TIFF) [file pbio.3000192.s009.tiff]

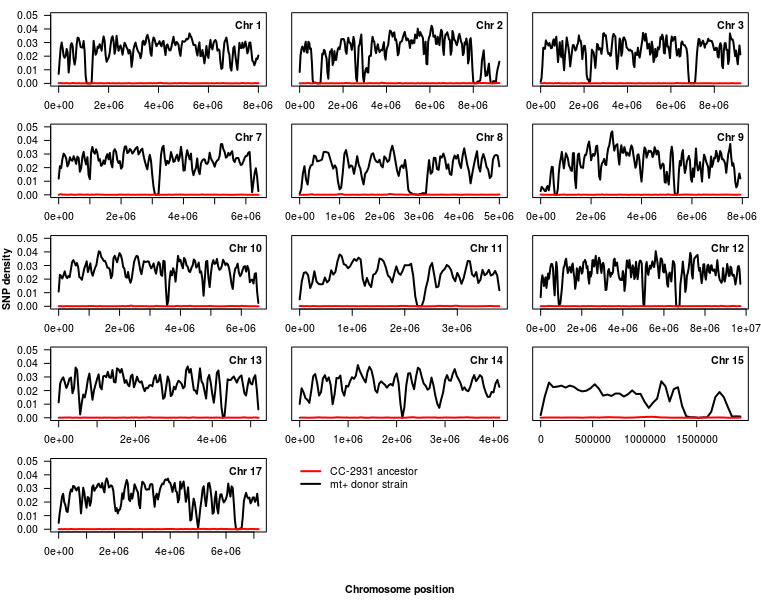

Supplement: S10 Fig — SNP densities were calculated for 80-kb windows along the chromosomes between the compatible ancestor and CC-2931 (red) and between the compatible ancestor and the mating type + donor strain (black). A mutation density of 0 indicates no genetic differences between the compatible ancestor and the strain it was compared to. Underlying data for this figure can be found in S9 Data. (TIFF) [file pbio.3000192.s010.tiff]

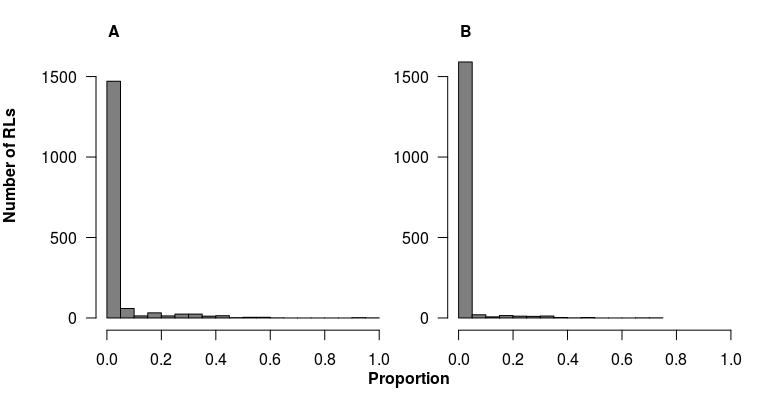

Supplement: S11 Fig — The distribution of (A) the proportion of missing data, i.e., noncallable mutations across the whole data set, and (B) the proportion of heterozygous calls. Based on these distributions, RLs with >10% missing data and/or >5% heterozygous calls were excluded from all analyses. Underlying data for this figure can be found in S10 Data. (TIFF) [file pbio.3000192.s011.tiff]

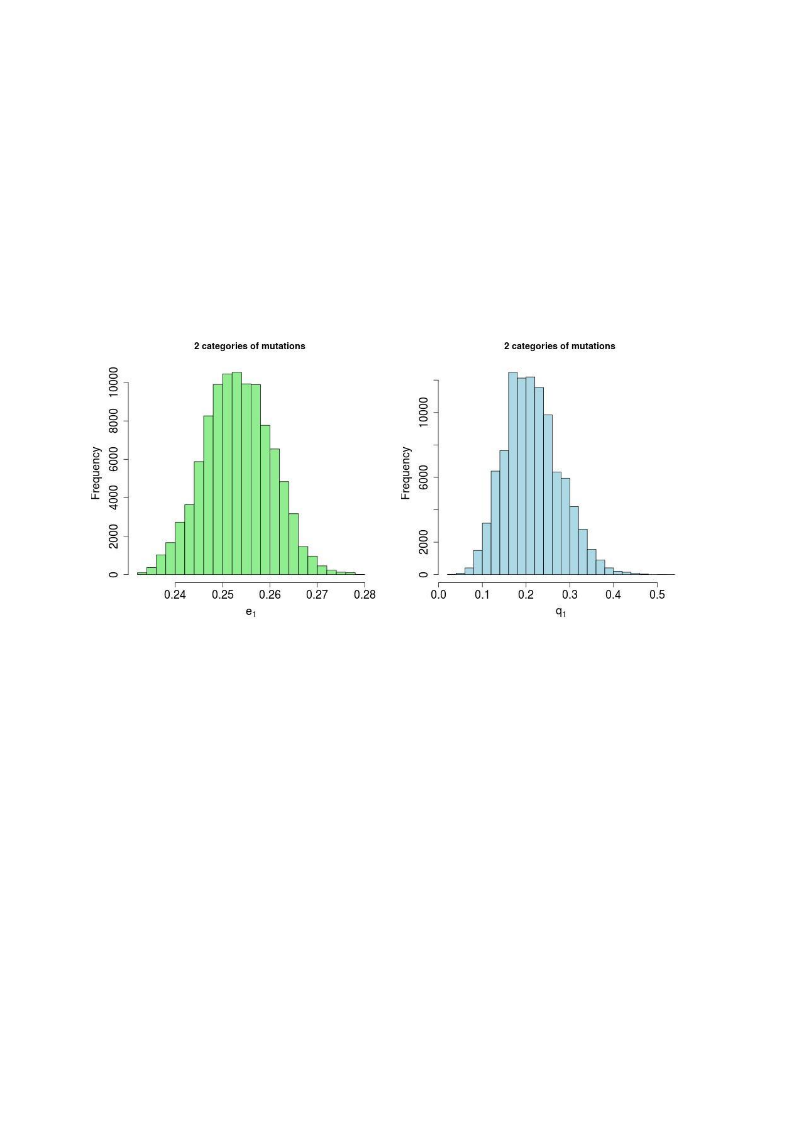

Supplement: S12 Fig — The simulated values were e1 = 0.25 and q1 = 0.2. The mutation effects here and in S2 and S3 Figs are expressed in phenotypic standard deviation units. There were 40 mutations simulated and 10,000 observations. Software and commands underlying this figure can be found in S4 Data. MCMC, Markov chain Monte Carlo. (TIF) [file pbio.3000192.s012.tif]

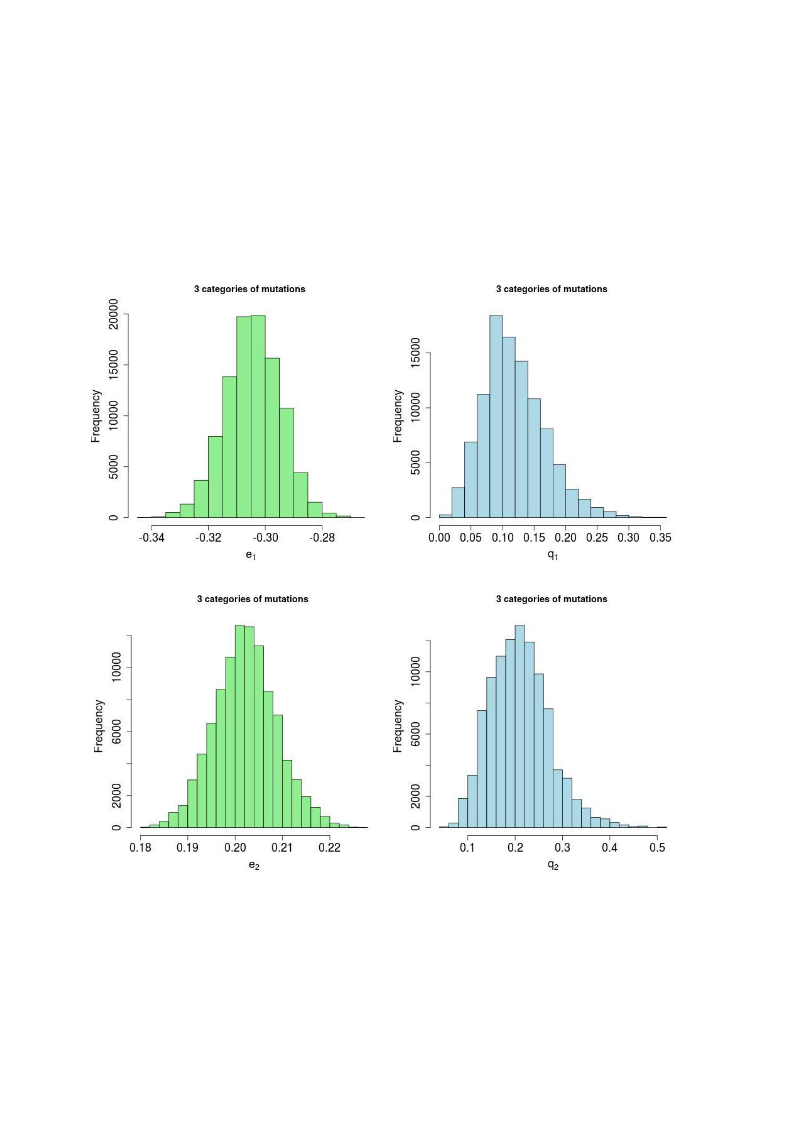

Supplement: S13 Fig — The simulated values were e1 = −0.3, q1 = 0.1, e2 = 0.2, and q2 = 0.2. Software and commands underlying this figure can be found in S4 Data. MCMC, Markov chain Monte Carlo. (TIF) [file pbio.3000192.s013.tif]

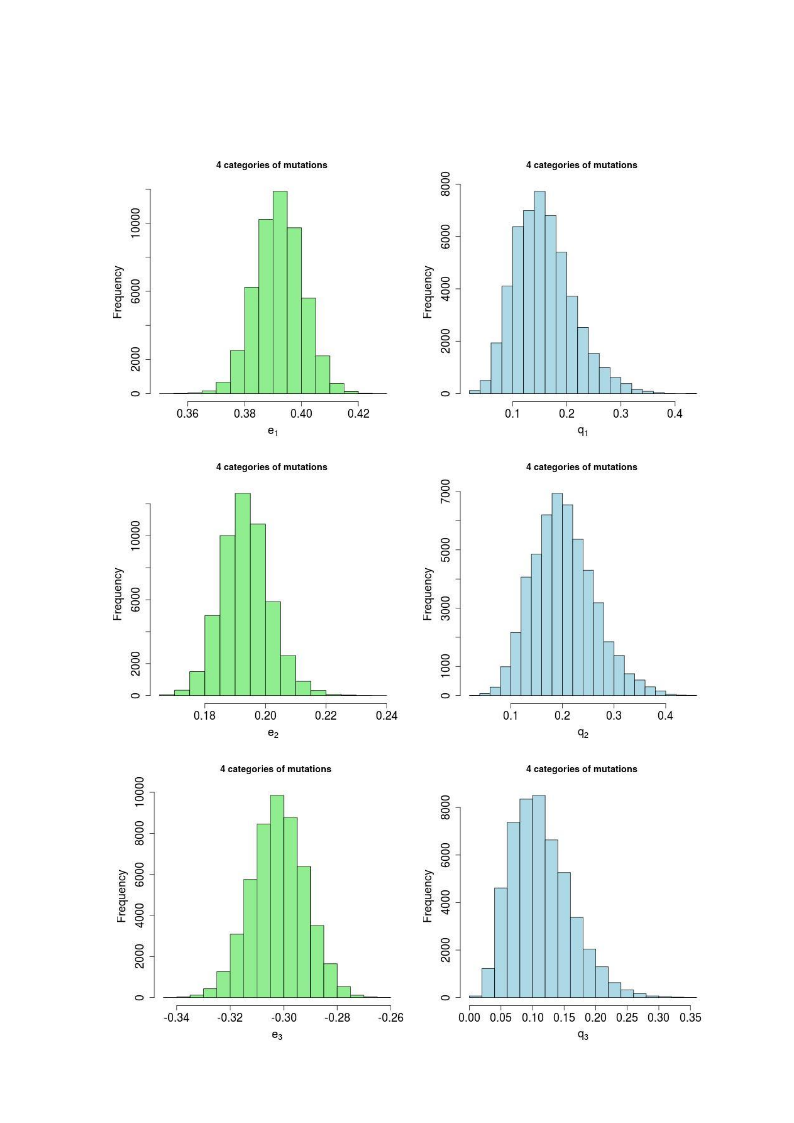

Supplement: S14 Fig — The simulated values were e1 = 0.4, q1 = 0.15, e2 = 0.2, q2 = 0.2, e3 = −0.3, and q3 = 0.1. Software and commands underlying this figure can be found in S4 Data. MCMC, Markov chain Monte Carlo. (TIF) [file pbio.3000192.s014.tif]

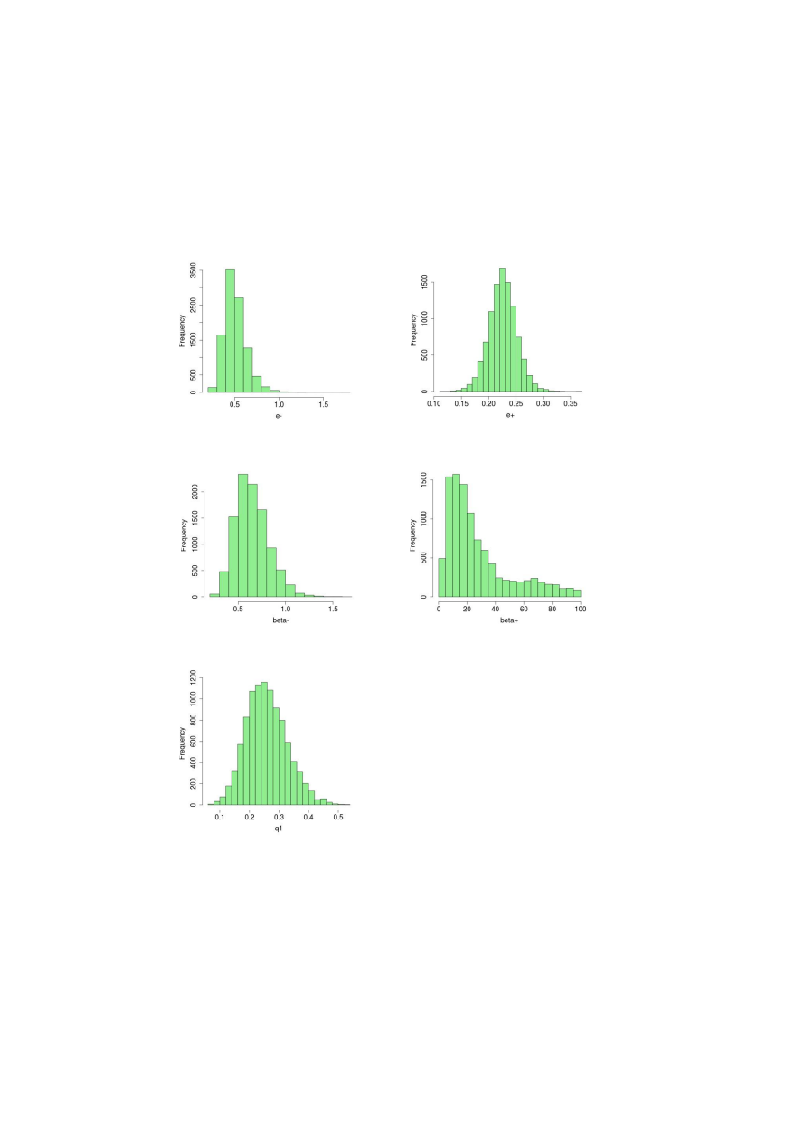

Supplement: S15 Fig — The simulated values were e− = 0.5, e+ = 0.25, beta− = 0.5, beta+ = 2, q1 = 0.25. Software and commands underlying this figure can be found in S4 Data. MCMC, Markov chain Monte Carlo. (TIF) [file pbio.3000192.s015.tif]
