# Supplementary material for: Inferring the distribution of fitness effects of spontaneous mutations in Chlamydomonas reinhardtii
Source: PLoS Biol. 2019 Jun 26;17(6):e3000192. doi: 10.1371/journal.pbio.3000192 (PMC6615632; doi:10.1371/journal.pbio.3000192)
Supplement: S5 Table — (DOC) [file pbio.3000192.s020.doc]

|  | ***Parameter estimate (95% credible interval)*** | |
| --- | --- | --- |
| ***MA Line*** | ***e_1_*** | ***q_1_*** |
| L03 | -0.018 (-0.040, -0.003) | 0.127 (0.031, 0.693) |
| L06 | 0.001 (-0.049, 0.060) | 0.017 (0.003, 0.729) |
| L07 | -0.019 (-0.063, 0.040) | 0.019 (0.005, 0.765) |
| L09 | 0.001 (-0.043, 0.044) | 0.002 (0.001, 0.819) |
| L11 | 0.051 (0.012, 0.072) | 0.032 (0.007, 0.181) |
| L14 | -0.038 (-0.061, -0.016) | 0.051 (0.016, 0.192) |
